# Astrocytic cell adhesion genes linked to schizophrenia correlate with synaptic programs in neurons

**DOI:** 10.1016/j.celrep.2022.111988

**Published:** 2023-01-12

**Authors:** Olli Pietiläinen, Aditi Trehan, Daniel Meyer, Jana Mitchell, Matthew Tegtmeyer, Vera Valakh, Hilena Gebre, Theresa Chen, Emilia Vartiainen, Samouil L. Farhi, Kevin Eggan, Steven A. McCarroll, Ralda Nehme

**Affiliations:** 1 Stanley Center for Psychiatric Research, Broad Institute of Harvard and MIT, Cambridge, MA 02142, USA; 2 Department of Stem Cell and Regenerative Biology and the Harvard Institute for Stem Cell Biology, Harvard University, Cambridge, MA 02138, USA; 3 Neuroscience Center, Helsinki Institute for Life Science, University of Helsinki, 00290 Helsinki, Finland; 4 Klarman Cell Observatory, Broad Institute of MIT and Harvard, Cambridge, MA 02142, USA; 5 Centre for Gene Therapy and Regenerative Medicine, King’s College, London WC2R 2LS, UK; 6 Department of Genetics, Harvard Medical School, Boston, MA 02115, USA; 7 Senior author; 8 Lead contact

## Abstract

The maturation of neurons and the development of synapses, although emblematic of neurons, also relies on interactions with astrocytes and other glia. Here, to study the role of glia-neuron interactions, we analyze the transcriptomes of human pluripotent stem cell (hPSC)-derived neurons, from 80 human donors, that were cultured with or without contact with glial cells. We find that the presence of astrocytes enhances synaptic gene-expression programs in neurons when in physical contact with astrocytes. These changes in neurons correlate with increased expression, in the cocultured glia, of genes that encode synaptic cell adhesion molecules. Both the neuronal and astrocyte gene-expression programs are enriched for genes associated with schizophrenia risk. Our results suggest that astrocyte-expressed genes with synaptic functions are associated with stronger expression of synaptic genetic programs in neurons, and they suggest a potential role for astrocyte-neuron interactions in schizophrenia.

## In brief

Pietiläinen et al. examine gene-expression changes in neuronal and glial cells on contact. By studying 80 human neuronal lines cultured in different formats, they find that synaptic gene-expression programs are enhanced in neurons, whereas synaptic cell adhesion programs are increased in glia. Both are enriched for schizophrenia risk genes.

## Graphical Abstract

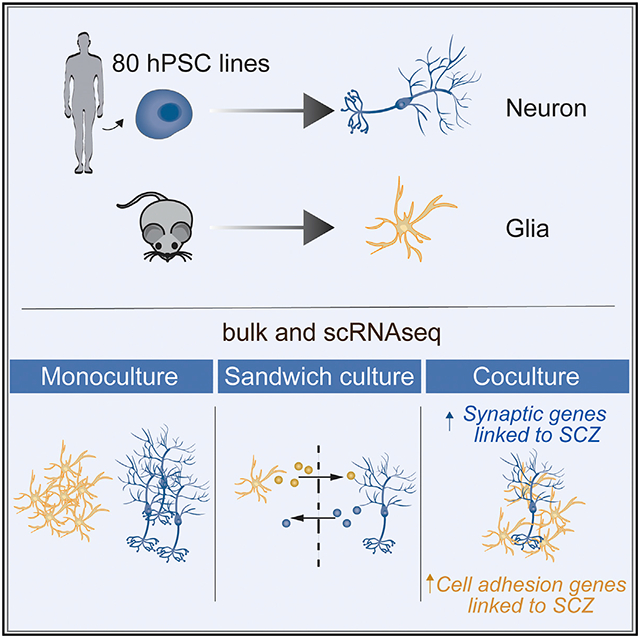

## INTRODUCTION

Schizophrenia is a severe brain disorder characterized by delusions and hallucinations, impairments in executive and other cognitive function, and flattened motivation, emotion, and interest.^1^ It affects 1% of people globally, but the biological mechanisms underlying the disorder are unknown.^2^ Inheritance is a major risk factor, and recent genetic discoveries have highlighted the quantitative enrichment of genes that are highly expressed by neurons and encode proteins that function at synapses.^3-5^ These fundamental aspects of neuronal biology are dependent on interactions with glial cells, including astrocytes,^6,7^ and raise the question whether cell-nonautonomous effects of glial cells on neurons are relevant to brain disorders such as schizophrenia.

Astrocytes provide neurons with homeostatic support and regulate neuronal development and maturation.^7^ They participate in the formation and shaping of the neuronal network by regulating synapse generation and elimination, transmission, and plasticity.^6,8,9^ Astrocytes surround neuronal cell bodies and synapses and interact with neurons through a range of contact-dependent and secreted signals that contribute to neuronal maturation.^8,10,11^ However, although glial cells are necessary for the functional maturation of neurons,^12-15^ many gaps remain in our understanding of the specific cellular and molecular programs that mediate these processes. To investigate the molecular pathways underlying glia-induced neuronal maturation, we performed RNA sequencing (RNA-seq) of human pluripotent stem cell (hPSC)-derived excitatory neurons that were cocultured with mouse glial cells. We reasoned that cellular responses to regulatory interactions between neurons and glia would be in part mediated through mutual changes in cell states that could be detected as correlated gene expression in the two cell types.^16,17^ The cross-species cell culture enabled us to separate transcripts by their origin and, therefore, identify molecular dependencies between cocultured neurons and glial cells by studying joint variability in their transcript abundances. Our analysis revealed that astrocyte-expressed synaptic cell adhesion molecules, including *NRXN1*, with established roles in schizophrenia were associated with induction of synaptic genetic programs in cocultured neurons. We further found that these genes were induced in the glial cells upon coculture with relevant to schizophrenia and provide insight into the potential role of astrocytes in psychiatric disorders.

## RESULTS

### Transcriptional profiling of hPSC-derived neurons grown with or without glial cells

**Figure 1.**
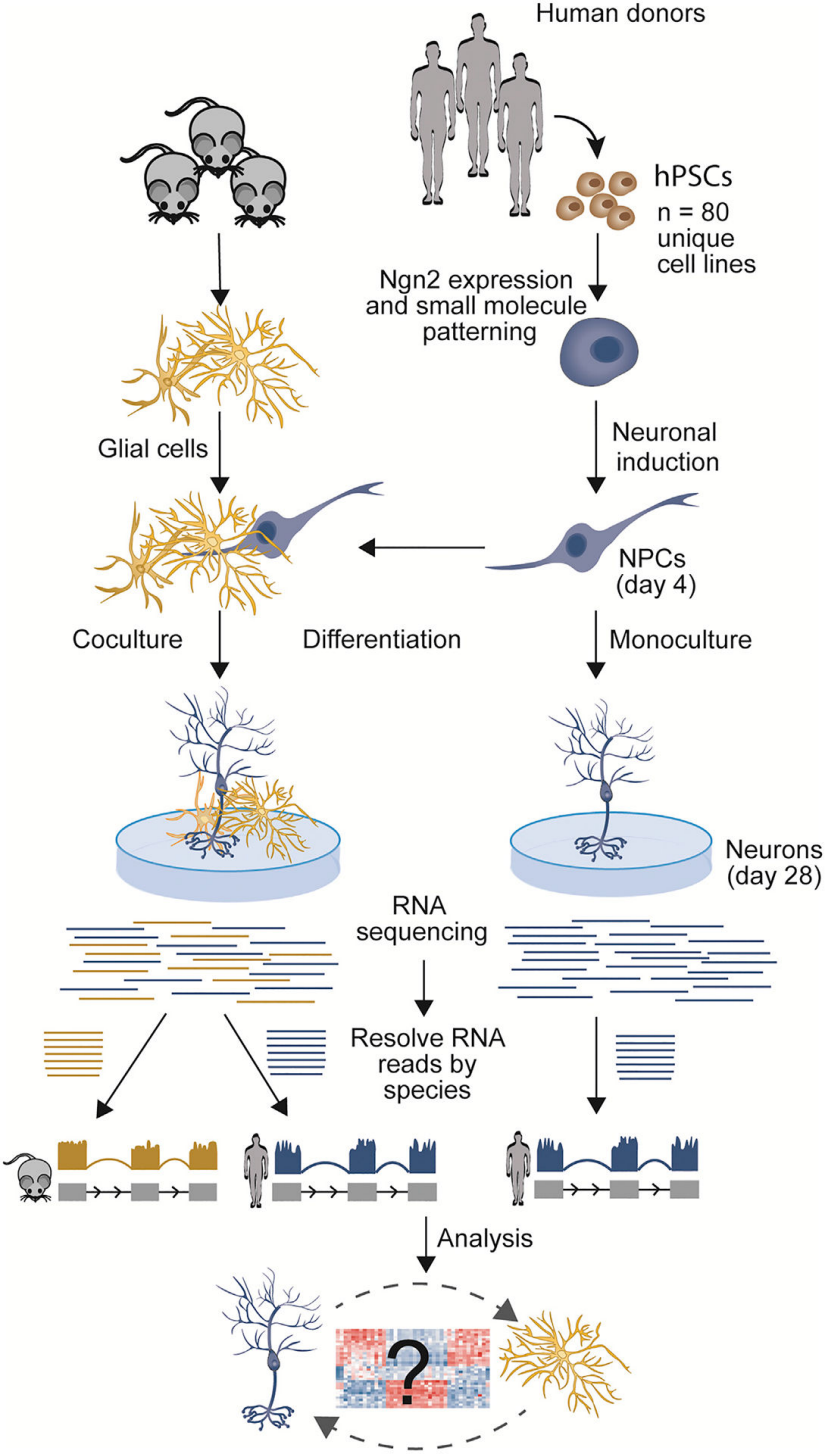
Outline of the experimental design Neuronal induction with Ngn2 and small molecule patterning was conducted for hPSCs from 80 healthy donors. At day 4 of the differentiation, part of the neuronal cultures was plated on mouse glial cells and others were left to differentiate alone. RNA-seq was performed at day 28 of the differentiation, and the libraries were resolved by species followed by analysis of normalized read counts.

**Figure 2.**
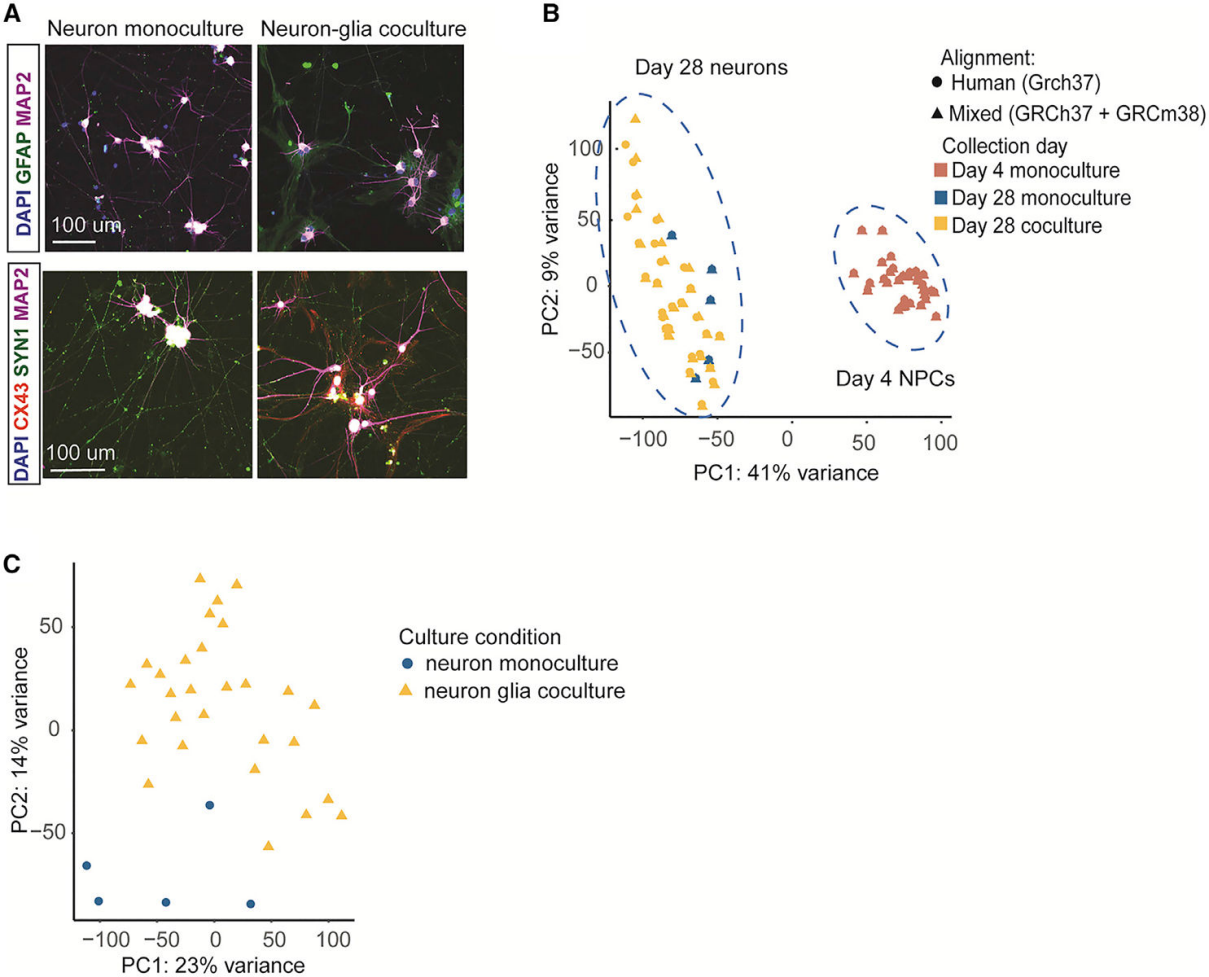
Characterization of human neurons (A) Representative images of neuron monocultures and neuron glia cocultures stained for (top) DAPI (blue), GFAP (green), and MAP2 (magenta) and (bottom) DAPI (blue), CX43 (red), Syn1 (green), and MAP2 (magenta). Scale bars, 100 μm. (B) PCA of RNA-seq data from day 4 NPCs (n= 34) and day 28 neurons (n = 32) aligned to human and mixed reference genomes. (C) PCA of RNA-seq data from day 28 neurons aligned to mixed reference genome (n = 32).

To study molecular pathways in glia-neuron interactions, we first assembled a sample set of 32 karyotypically normal hPSC lines (29 induced pluripotent stem cell [iPSC] and 3 embryonic stem cell [ESC] lines) derived from neurotypical donors^12,18^ (Figure 1; Table S1). These lines were differentiated into excitatory neurons using a well-characterized protocol that combines Ngn2 expression with forebrain patterning factors to robustly generate a homogeneous population of excitatory neurons (n = 32 cell lines).^12^ At day 4, we plated the neuronal cultures onto a monolayer of mouse glial cells that were prepared as described previously^19^ from the cortex of neonatal mice (post-natal day [P] 1 to P3). We have previously shown that coculture with glial cells is necessary for functional neuronal maturation, which we assessed by measuring synaptically driven network activity using multielectrode arrays (MEAs).^12^ To study the effect of glia coculture on neurons, we first compared the cocultured neurons with a set of five hPSC lines (out of 32) that were differentiated in glia-free condition. In the absence of glia, neuronal cells tended to cluster and clump up together, whereas neurons cultured with glia appeared overall healthier and were more evenly distributed on the glial monolayer (Figures 2A and S1A). Consistent with previous work, this showed that the glia coculture is overall beneficial for neurons, and that physical contact with neurons was required to induce many of the pro-synaptic effects. These data suggest that the cellular processes in glia that are associated with neuronal maturation involving synaptic programs *in vitro* are the neurons. We then collected RNA-seq data from the 32 cultures at day 28 of the experiment, a point at which the neurons cocultured with glia display robust neuronal morphology and electrophysical activity.^12^ To explore differences in developmental trajectories, we also performed RNA-seq at day 4 of the differentiation for the same lines (in 34 independent differentiations) prior to glia coculture, when the differentiating cells resembled neuronal progenitor-like cells (NPCs),^12,20^ totaling to 67 RNA libraries (Table S1). All 67 differentiations were carried out in triplicates.

### Leveraging cross-species coculture to distinguish RNA molecules from different cell types

To characterize cell-type-specific transcriptional effects in the two-species coculture, we first confirmed that we could reliably distinguish RNA molecules that originated from the human neurons from those that originated from the mouse glial cells.^21,22^ We aligned the reads to a combined Ensembl human-mouse reference genome (GRCh37/hg19 and GRCm38/mm10, respectively; GEO: GSE63269)^22^ and compared these with read counts obtained after aligning to human genome assembly alone (GRCh37/hg19). We used variance partitioning to estimate the proportion of variability in the gene-expression estimates arising from differences between the two alignments. We found minimal cross-species mapping. For most genes, the differences added little or no variance to the RNA abundance estimates (mean = 0.78%, median = 0% of total variance explained). Importantly, only for 329 of 19,185 genes did the alignment have effects that reached 10% of the inter-sample variance in gene expression (Figure S1B). These 329 genes were on average 22.3 kb shorter than the rest (95% confidence interval [CI]: 14.1–30.6 kb; p = 0.00035, Mann-Whitney) and did not have any specific Gene Ontology (GO) enrichment (q > 0.05 for all terms). As expected, the alignment to the mixed genome yielded slightly lower (average 3.9%) library size because of reads that mapped ambiguously between the two species’ genomes (average pairwise difference in read counts = 506 × 10^3^ reads [95% CI: 765 × 10^3^ to 247 × 10^3^], p = 0.0002, paired t test).

A principal-component analysis (PCA) of the RNA expression profiles of the day 28 neurons and day 4 NPCs confirmed that the alignment effect was similar in the coculture and monoculture experiments and did not confound the primary sources of biological variability in the data (Figure 2B). As evident from the superimposed data in PCA, the primary components of gene expression were not affected by the alignment. Instead, the small fraction of genes whose expression estimates were impacted by cross-species mapping were captured in principal components (PC) 7 and PC8 (explaining 5% of the total variance) (Figure S1C). Importantly, this was not dependent on the differentiation stage or the presence of glia coculture, suggesting that there was no major bias by experimental condition in the read count estimates. Together, these results confirmed that we could accurately distinguish gene expression in human neurons from that in cocultured mouse glial cells.

### Neurons grown with mouse glia cells exhibit global changes in gene expression

Having confirmed that we could reliably assign RNA sequence reads to their cell population of origin, we next sought to confirm the neuronal identity of the day 28 neurons (Figure S1D). A comparison of the expression of canonical marker genes (Table S2) in neurons and day 4 NPCs confirmed the expected temporal reduction of the pluripotency genes *POU5F1*/Oct4 and *MKI67* (neuron monoculture: β = −1.48, SE: 0.24, p = 7.95 × 10^−9^; coculture: β = −1.39, SE: 0.13, p = 5.4 × 10^−20^). Neuronal identity genes (*DCX, MAP2, MAPT, NCAM1, RBFOX3, SYN1, TUBB3*) were induced over time in day 28 neurons compared with day 4 NPCs (monoculture: β = 0.36, SE: 0.17, p = 0.03; coculture: β = 0.75, SE: 0.09, p = 3.1 × 10^−16^), whereas neuronal progenitor markers genes (*EMX2, HES1, MSI1, NEUROD1, OTX2*) were slightly reduced (neuron monoculture: β = −0.46, SE: 0.20, p = 0.03; coculture: β = −0.42, SE: 0.11, p = 0.0002).

We then focused on characterizing the global transcriptome landscape of the differentiating neurons in both conditions. As expected, the largest component of variation in PCA (PC1: 44%) reflected differentiation/maturation time (day 4 versus day 28 samples). Interestingly, within the neuron cluster, neurons grown as a monoculture separated from neurons in coculture and grouped slightly closer to NPCs (Figures 2C and S1E). Taken together, the data suggested that glia coculture induced global transcriptional changes consistent with greater neuronal maturation. Furthermore, we found that culture-to-culture variability of the cocultured neurons was markedly larger (dispersion: mean = 0.27, median = 0.21) than in neurons in monoculture (dispersions: mean = 0.17, median = 0.11) and in NPCs (dispersions: mean = 0.16, median = 0.12; Figure S1F). This suggested that the glial cells introduced an additional source of variance into the neuronal system. We reasoned that this variance could be, at least in part, the result of differences in the glial cell state and composition in the primary mouse cultures and could therefore inform the study of biological drivers underlying glianeuron interaction.

### Astrocytes are the predominant cell type in the mouse glial cultures

**Figure 3.**
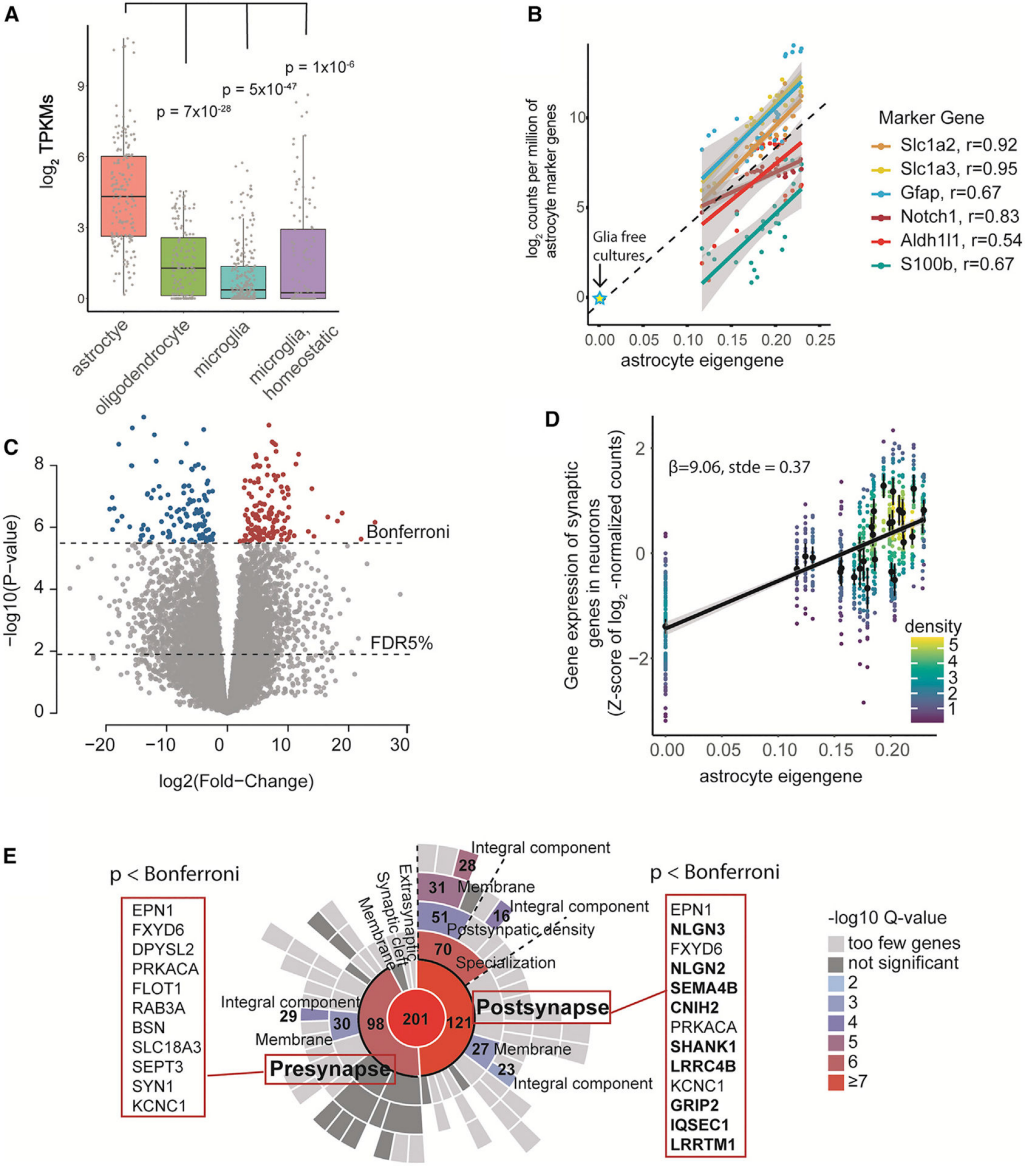
The astrocyte eigengene associates with synaptic gene expression in cocultured neurons (A) Expression of canonical markers for glial cells in the cocultures (n = 28). The glia-cocultures have higher expression of astrocyte marker genes than marker genes for oligodendrocytes (p = 7 × 10^−28^), microglia (p = 4 × 10^−47^), and homeostatic microglia (p = 1 × 10^−6^). The boxplot is in Tukey style (Q1, Q2, Q3), and the whiskers present the minimum and maximum. (B) Astrocyte marker genes are highly correlated with the astrocyte eigengene (n= 32). (C) Astrocyte eigengene values associate with the expression of 4,195 genes (1,970 induced, FDR < 5%, 250 were transcriptome-wide significant, p < 3.0 × 10^−6^, Bonferroni, colored genes, n= 32). (D) Synaptic genes were induced in neurons in cocultures with high expression of astrocyte marker genes. (E) Astrocyte induced genes in neurons were enriched for synaptic annotations in SynGO.

Next, we moved on to characterize the glial cell population in the 28 cocultures by exploring the expression of genes characteristic of specific glia cell types from the mouse (Table S2). In line with previous work,^19^ we observed predominant expression of genes characteristic of astrocytes (mean: 4.4 log_2_TPKM; transcripts per kilobase per million) compared with marker genes for oligodendrocytes (Δ = 2.9 log_2_TPKM, p = 7 × 10^−28^), homeostatic microglia (Δ = 1.0 log_2_TPKM, p = 1 × 10^−6^), and microglia (Δ = 3.5 log_2_TPKM, p = 5 × 10^−47^) in the glia coculture (Figure 3A).

Guided by our observation of increased variability in cocultured neurons, which we presumed to result from biological variability in glial cell composition and state, we decided to investigate whether the transcriptional profile of the glial cell population in coculture was associated with neuronal maturation state. Because astrocytes have been found to regulate neuronal maturation,^23^ we specifically sought to determine whether genetic programs related to astrocytes were associated with differences in the maturation. We first estimated the relative abundance of astrocytes in each of the 28 cocultures by combining information from six canonical astrocyte marker genes (*Aldh1l1, Slc1a3, Slc1a2, Gfap, Notch1, S100b*). We used singular value decomposition (SVD) to generate a single astrocyte eigengene from the marker genes distinctive of astrocytes (Figure 3B; Table S1). The marker genes were highly correlated, and the eigengene captured 97% of the variance in the glial cell composition and cell state in the culture. This is consistent with the marker gene transcripts originating from a single shared cell type,^24^ presumed to be astrocytes, in the glia coculture, and further confirmed the generated eigengene as a relevant proxy for astrocytes in the glial coculture. To measure the full effect of glia-neuron coculture on gene expression, we included the five glia-free monocultures (out of 32) as the negative control for glia-induced outcomes in neurons. For glia-free cultures, the astrocyte eigengene value was set to zero. This was consistent with an extrapolated value (0.008) from the astrocyte marker gene expression for the glia-free cultures (Figure 3B). We hypothesized that by leveraging a range of quantitative effects across many cultures, we could also better control for the observed qualitative culture differences between the monocultures and cocultures.

### Astrocytes induce transcripts with synaptic functions in neurons

To investigate whether this gene-expression variation in the astrocytes (across the individual cultures) was associated with gene-expression variation in the neurons, we used a multifactorial linear model (Limma-voom^25^) to model each gene’s neuronal expression level, with the astrocyte eigengene and normalized *Ngn2* expression as explanatory variables. The analysis revealed transcript abundances of 4,195 human genes (out of 16,694) to be associated (at false discovery rate [FDR] < 5%) with the astrocyte eigengene value. These included 1,970 positive and 2,225 negative associations to the astrocyte eigengene (Figure 3C; Table S3). Overall, the astrocyte eigengene explained 4%–70% of variance in the 4,195 significantly changed genes (median 21%), while the level of the neuralizing *Ngn2* and residual sources of variance also had a marked contribution (median: 11% and 67%, respectively) to the gene-expression levels (Figure S2A).

To explore whether the genes whose neuronal expression levels were associated with high astrocyte eigengene values were enriched for specific biological functions, we used GO enrichment analysis, focusing on 250 genes (135 induced, 115 reduced) that were associated with the coculture astrocyte eigengene at a still-higher level of significance (p < 3.0 × 10^−6^, Bonferroni) (Figure 3C; Table S3). The analysis of the 135 transcriptome-wide significantly induced neuronal genes revealed most significant enrichments for functions in synapse assembly (GO:0007416, n = 9 genes, fold change [FC] = 7.03, q = 0.004), axon development (GO:0061564, n = 15 genes, FC = 3.5, q = 0.005), negative regulation of the JAK-STAT cascade (GO:0046426, n = 5, FC = 13.6, q = 0.005), and chemical synaptic signaling (GO:0007268, N = 16, FC = 3.2, q = 0.005) (Figure S2B; Table S4). This suggested that astrocytes in the cocultures induced genetic programs related to synapse biology in the neurons. Interestingly, among the most frequently included genes in the enriched categories were genes encoding transsynaptic cell adhesion proteins, including neuroligins (*NGLN2, NGLN3, NECTIN1*) and leucine-rich repeat membrane proteins (*LRRC4B, LRRTM1*), with roles in anchoring the synaptic nerve terminals. In comparison, the 115 transcriptome-wide significantly reduced neuronal transcripts in response to astrocytes did not reveal significant enrichment for any specific biological process.

We further asked whether the larger set of 1,970 induced genes (that passed the FDR < 5% threshold) were enriched for specific synaptic components in SynGO.^26^ We observed a significant enrichment for genes in the presynaptic component (GO:0045202, n = 98, genes, q = 8.4 × 10^−7^) and for postsynaptic genes (GO:0098794, n = 121 genes, q = 2.9 × 10^−8^), especially in postsynaptic specialization (n= 70 genes, q = 3.6 × 10^−7^) and postsynaptic density (n = 51 genes, q = 2.1 × 10^−4^) (Figures 3D and 3E; Table S5). These included 20 transcriptome-wide significantly induced neuronal genes, 13 of which were postsynaptic (Figure 3E). Together these results implied that astrocytes in coculture induce or enhance genetic programs in neurons related to pre- and postsynaptic biology, which is consistent with synaptic maturation.

### Replication in isogenic cultures

**Figure 4.**
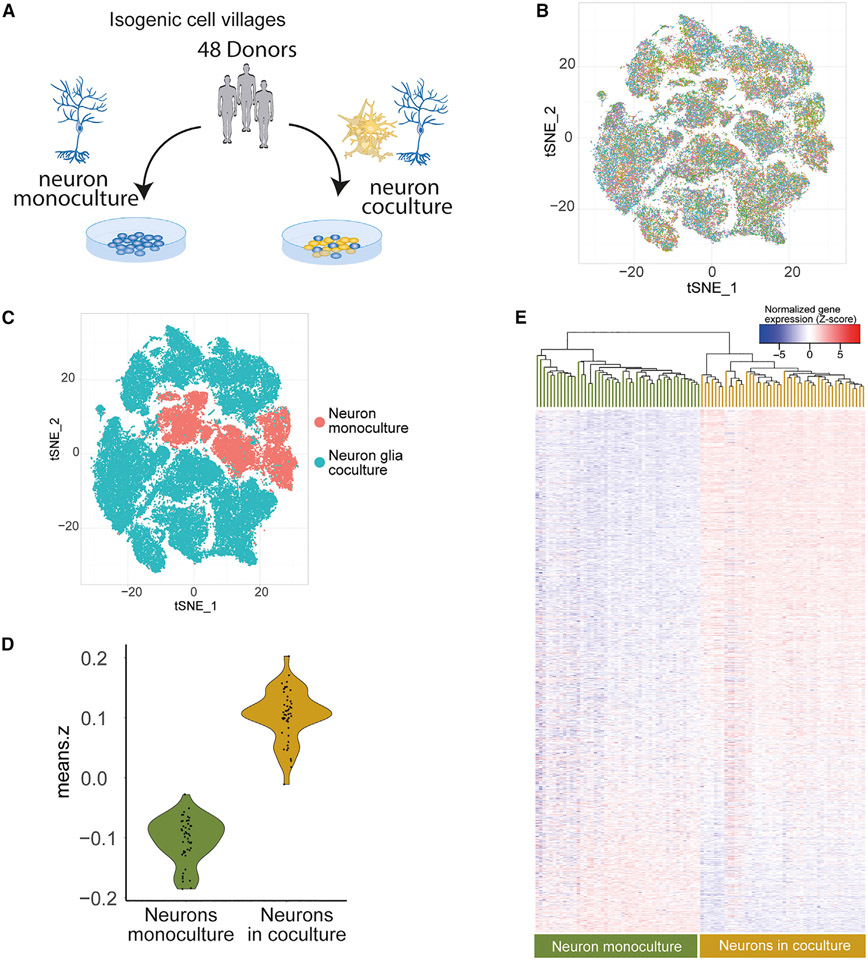
Cell villages of isogenic neurons grown in monoculture or in coculture (A) Study design for scRNA-seq of pooled neuron villages from 48 donors (in total 96). (B) t-SNE projection of scRNA-seq data color-coded by donor. (C) t-SNE projection of scRNA-seq data color-coded by culture condition (neuron monoculture or coculture with glia). (D and E) Neurons in coculture have higher expression of genes associated with the astrocyte eigengene (difference: 0.21 standard deviations, 95% CI: 0.22–0.19, p = 1.45 × 10^−43^). The genes are ordered based on the magnitude of log_2_fold-change from largest to smallest. The gene labels are in Table S7.

We next sought to evaluate whether the 1,970 neuron-expressed genes that were correlated with the high astrocyte eigengene values in the accompanying glial cells were induced in neurons by coculture. We generated pooled cell cultures from an additional 48 donors that were differentiated into neurons either in the presence or absence of glial cells. Neurons from each of the 48 donors were differentiated together in a “cell village” as previously described.^27,28^ In brief, neurons from each of the 48 cell lines were induced separately, then mixed in equal numbers 6 days post-induction to form a “village” and differentiated together either with or without glia (Figure 4A). At day 28 of the differentiation, we performed droplet-based single-cell RNA-seq (scRNA-seq) on the neuron villages using the “Dropulation” method, which uses natural donor SNP genotypes as allelic barcodes to assign donor identity for each cell. The pooled experiment enabled the generation of cell cultures from multiple donors that were harmonized for identical culture conditions. The cell villages yielded RNA-seq data from 76,112 cells with, on average, 793 cells from each donor and, on average, 15,586 unique molecular identifiers (UMIs) per cell (Table S6). We used t-distributed stochastic neighbor embedding (t-SNE) to explore the impact of the glial cell on the state of the cocultured neurons (Figures 4B and 4C). The t-SNE demonstrated an even distribution of cells from all donors across the dataset, demonstrating that the cell villages were overall well balanced, and highlighted the reproducibility of the differentiation protocol. Furthermore, we could see that the monocultured neurons clustered separately from the cocultured neurons. This was in line with the PCA from the bulk RNA-seq from the discovery cohort. We then constructed meta cells of each donor and compared the 1,970 transcript that were associated with high astrocyte eigengene values between the isogenic neuron villages that were grown with or without the glial cells. Reassuringly, we found that these transcripts had in aggregate higher abundance in the cocultured village than in the glia-free neuron village (difference: 0.21 standard deviations, 95% CI: 0.22–0.19, p = 1.45 × 10^−43^, t test; Figure 4D). Of the 1,626 genes expressed in both datasets, 65% (1,088) were expressed more highly by neurons in glia coculture than in neuronal monoculture, with 78% (846) of these differences being nominally significant (FDR < 5%; p = 7.0 × 10^−9^, binomial test) (Figures 4E and S3A; Table S7). In summary, these results confirmed that many of the transcripts that were found to associate with high astrocyte eigengene values in the initial experiments were also induced in neurons in glia cocultures in independent experiments. This is consistent with the idea that astrocyte biology instructs, rather than merely responds to, the neuronal changes.

### Neurons induce the expression of genes for cholesterol synthesis and synaptic functions in astrocytes

**Figure 5.**
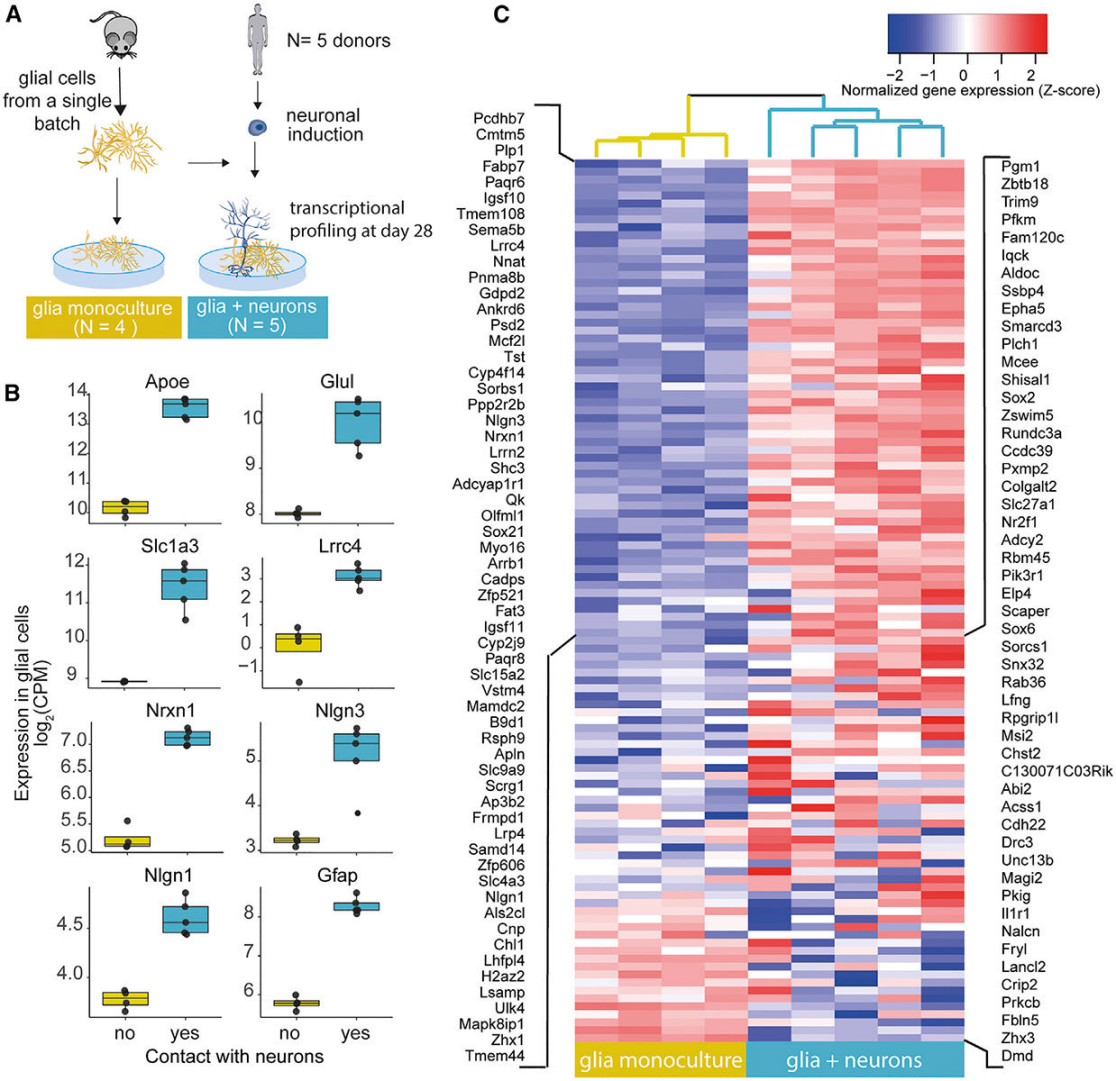
Neuronal impact on glial gene programs (A) Schematic of the experimental design. Gene expression of glial cells was compared in glia monocultures (n = 4) and in coculture (n = 5) with day 28 neurons. (B) Neuronal coculture-induced expression of genes in glial cells that were associated with neuronal synaptic maturation. (C) Neuron presence induces expression of transcripts of synaptic members, including synaptic cell adhesion molecules.

Having established that glia cocultures that have high expression of astrocyte marker genes associate with induction of synaptic gene programs in accompanying neurons, we wondered what biological processes in astrocytes underlie these effects. To address this, we first generated RNA-seq data from glial cells in coculture with neurons at day 28 of neuron differentiation (n= 5, 4 replicates each) and from glial cell monocultures of the same batch cultured at the same time (n = 4; Figure 5A). The presence of neurons resulted in substantial changes in astrocyte gene expression, with 1,426 (out of 11,205 evaluated) genes differing in expression between the coculture and monoculture conditions (p < 4.5 × 10^−6^, Bonferroni; 668 induced in coculture with neurons; Table S8). The enhanced transcripts were particularly enriched for genes with functions in the cholesterol biosynthetic process (GO:0006695; n = 15 members, 7.8 times more genes relative to background, q = 6.3 × 10^−8^; Figure S4; Table S9). The induction of the cholesterol synthesis is consistent with previous reports suggesting that astrocytes are a major provider of neuronal cholesterol. The most significantly induced genes included *Apoe* and Clu (ApoJ), which encode for lipoproteins that shuttle cholesterol from astrocytes to neurons (log_2_-fold-change = 3.4, p = 1.8 × 10^−14^, log_2_fold-change = 1.4, p = 7.0 × 10^−11^, respectively; Figure 5B). In addition, coculture with neurons enhanced glial expression of several genes with annotated roles in synaptic physiology (GO:0099536; n = 48, 2.4-fold enrichment, q = 2.0 × 10^−6^). These included genes that encode the astrocyte-specific glutamate transporter (Slc1a3/Glast) and the glutamate-ammonia ligase (Glul) that catalyzes the conversion of glutamate, which is taken up from the synapse by astrocytes, to glutamine for recycling back to neurons (Figure 5B). These gene-expression changes align with the increase in glutamate-driven activity of the neurons that is observed in glia coculture, and they suggest that the glial cells actively participate in the glutamate cycle *in vitro*^12^ (genes encoding synaptic cell adhesion molecules, *Nrxn1, Lrrc4*, and *Nlgn3*, were also strongly upregulated in glial cells in the presence of neurons [*Nrxn1*: log_2_fold-change = 1.9, p = 4.5 × 10^−12^; *Lrrc4*: log_2_fold-change = 3.2, p = 2.3 × 10^−6^; *Nlgn3*: log_2_fold-change = 1.9, p = 8.4 × 10^−7^]; Figure 5B). Intriguingly, Nrxn1, Nlgn1, and Nlgn3 have recently been demonstrated to be abundant in astrocytes and likely have roles in the astrocyte processes that surround the synaptic contacts at the tripartite synapse.^29,30^ *Nlgn1* was also moderately upregulated in the cocultured glial cells (log_2_fold-change = 0.9, p = 2.1 × 10^−5^). In comparison, the genes whose expression was reduced by neuronal coculture were enriched for roles in developmental processes, cell structure, and motility (Table S10). Together these results demonstrated that glial cells undergo extensive changes in the cell state in response to neurons and suggested that astrocytes actively participate in neuronal biological processes *in vitro*.

### Astrocyte-expressed genes induce synaptic programs in neurons

**Figure 6.**
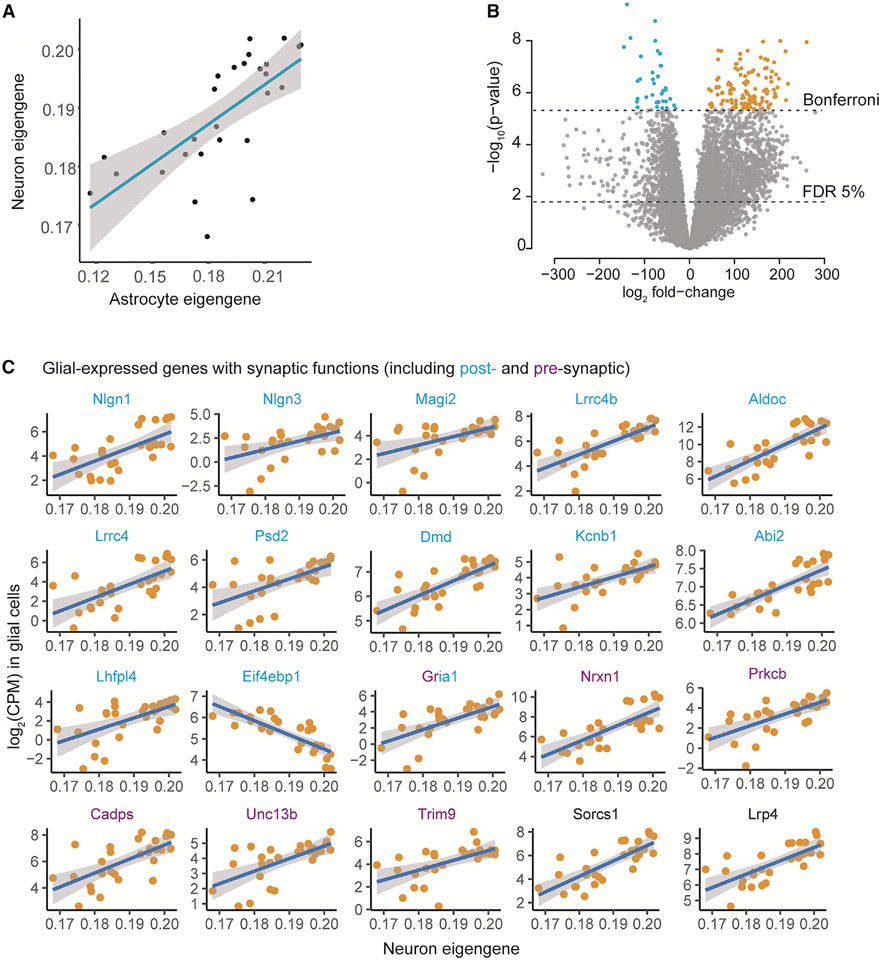
Differential expression of the neuron eigengene in glial cells (n = 28) (A) The neuron eigengene is correlated (r = 0.66) with the astrocyte eigengene values. (B) Expression of 159 glial genes is associated with the neuronal eigengene (4.3 × 10^−6^, Bonferroni). Differential expression analysis revealed 123 glial genes whose high expression is associated with neuronal maturation in coculture (orange) and 36 genes with negative association to the neuronal maturation state (blue). (C) Association of expression of glial genes with neuron eigengene. Postsynaptic genes are labeled in blue, presynaptic genes in purple, and other genes are in black.

We next asked whether these transcriptional changes in the glial cells were also associated with the synaptic maturation of neurons in coculture. We first condensed the neuronal expression profile of the 1,970 astrocyte-induced genes by SVD to a single eigengene in the discovery dataset (Table S1). The generated neuron eigengene values were similar to those of the 135 transcriptome-wide significantly astrocyte-induced genes in neurons (r_median_ = 0.65) and were highly correlated (r = 0.66) with the astrocyte eigengene, confirming that the neuron eigengene captured relevant variation in gene expression in the neurons (Figure 6A).

We then studied the association between the expression levels of individual glial genes (total of 11,627 mapped mouse genes) and the maturation state of the cocultured neurons defined by the neuron eigengene value. The analysis of the bulk RNA-seq in the discovery sample set revealed transcriptome-wide significant association of transcript levels of 159 glia genes with the neuron eigengene (p < 4.3 × 10^−6^, Bonferroni). Of these, 123 genes were positively correlated with the neuron eigengene of the more mature neuronal state, and 36 were negatively associated with the neuron eigengene values (Figure 6B; Table S11).

To gain insight into the likely source cell type of the 159 glia gene transcripts, we explored their expression in a scRNA-seq atlas of adult mouse brain.^31^ Comparison of the expression patterns of the meta cells from the brain atlas revealed that the 123 genes, whose high transcript levels in glia were associated with higher expression of neuron eigengene, were predominantly expressed by astrocytes compared with other glia cell types (p = 6.9 × 10^−26^, t test) (Figure S3B). In contrast, the 36 negatively associated glia transcripts showed no evidence of preferential expression between the glial cell types in the mouse brain (p = 0.81), and they were expressed at a significantly lower level in astrocytes than the 123 induced genes (p = 0.0035, t test) (Figure S3B). These results demonstrated that the individual genes associated with synaptic programs in neurons were abundantly expressed by astrocytes in the mouse brain and suggested a role in pathways related to astrocyte-neuron interaction.

### Expression of synaptic cell adhesion molecules by glial cells is associated with synaptic programs in neurons

We next asked whether these 159 glia genes reflected specific neurobiological processes that could underlie the interactions with neurons. GO term analysis of the 159 glia transcripts revealed an enrichment of genes with synaptic membrane functions (GO:0097060, n = 13 genes, FC = 4.4, q = 0.002) (Table S12). Importantly, this enrichment was driven by genes whose high expression in glial cells induced high expression of synaptic genes in neurons. Reduced expression of only one prosynaptic gene in glial cells, *Eif4ebp1*, encoding for Eukaryotic translation initiation factor 4e, was associated with high neuron eigengene values. Encouraged by these results, we explored curated synaptic annotations from SynGO to confirm 19 glia-expressed genes with annotation to synaptic component, of which 13 were postsynaptic (q = 0.007) and 6 presynaptic members (q = 0.25) (Figures 6C and S3C; Table S13). Remarkably, we noticed that many of these genes were synaptic cell adhesion molecules, including *Nrxn1, Nlgn1, Nlgn3, Lrrc4*, and *Lrrc4b*, similarly to what we found was induced in the glial cells after coculture with neurons. Further evidence for synaptic cell adhesion molecules came from the synaptic scaffold proteins Magi2 and Sorcs1, which associate with neuroligins and Nrxn1, respectively, in the synapse.^32,33^

Astrocytes secrete many molecules, including neurotransmitters, modulators, and trophic factors, via regulated exocytosis of synaptic-like vesicles.^34^ Interestingly, several of the identified presynaptic genes in the glia have annotated roles in regulating the synaptic vesicle cycle in SynGO (Cadps, Nrxn1, Unc13B, and Prkcb), suggesting a potential additional role for these genes in regulating the secretory vesicle cycle in glial cells. In addition, among the most significantly associated genes was the gene encoding the astrocytic low-density lipoprotein receptor-related protein Lrp4 (Figure 6C), which has roles in regulating glutamate transmission.^35^ Together these results suggested that genes encoding proteins with both pre- and postsynaptic functions and members of the tripartite synapse are expressed in glia cells and associated with induced expression of synaptic genetic programs in cocultured neurons.

In comparison, the astrocyte genes negatively associated with the neuronal eigengene did not reveal significant enrichment for specific biological processes. Interestingly, however, the most significantly reduced gene encoded for interleukin-6 family neuropoietic cytokine Lif (leukemia-inhibitory factor). Among its many roles, Lif promotes self-renewal and neurogenesis in neuronal stem cells, as well progression of astrocyte precursor cells to mature glial fibrillary acidic protein-positive (GFAP^+^) astrocytes.^36-38^ Therefore, the reduced Lif expression could indicate progression from neurogenesis/gliogenesis to more mature cell states. In line with this, *Gfap* was found to be significantly induced in the glial cells by coculture with neurons (log_2_fold-change = 2.5, p = 7.9 × 10^−14^; Figure 5B). Furthermore, high expression of *Cmmt5*, a marker of late astrocytes,^39^ was the gene most significantly positively associated to the neuronal eigengene. These results suggest that the maturing hPSC-derived neurons enhance the maturation of the accompanying glial cells.

### Neurons stimulate pro-synaptic gene-expression programs in glial cells

After identifying the set of 123 astrocyte-expressed genes whose high expression correlated with the maturation of the accompanying neurons, we examined whether these genes overlapped with those that were induced in the glial cells after coculture with neurons. Of the 123 genes, 111 were reliably detected in the glial cell monocultures, and 68 had significantly higher expression (adjusted p < 0.05) in glial cells in coculture (Figure 5C; Table S8). This overlap was highly unlikely to have occurred by chance (p = 5.5 × 10^−19^, binomial test). Of the 18 identified synaptic genes (Figure 6C), eight were induced in astrocytes in the presence of neurons (Figure 5C). These included all except for one of the synaptic cell adhesion molecules (*Nrxn1, Nlgn1, Nlgn2, Lrrc4*). Similarly, we found that of the 36 transcripts whose low expression in the glia coculture was associated with neuronal maturation, 16 were reduced in expression in the glial cells in the presence of neurons (Figure S3D). Taken together, our data suggested that glial cells undergo adaptive changes in response to differentiating neurons. These include induced levels of transcripts of synaptic cell adhesion molecules, whose expression in glial cells is in turn associated with advanced maturation of accompanying neurons in coculture.

### Physical contact with astrocytes promotes the induction of synaptic gene programs in neurons

**Figure 7.**
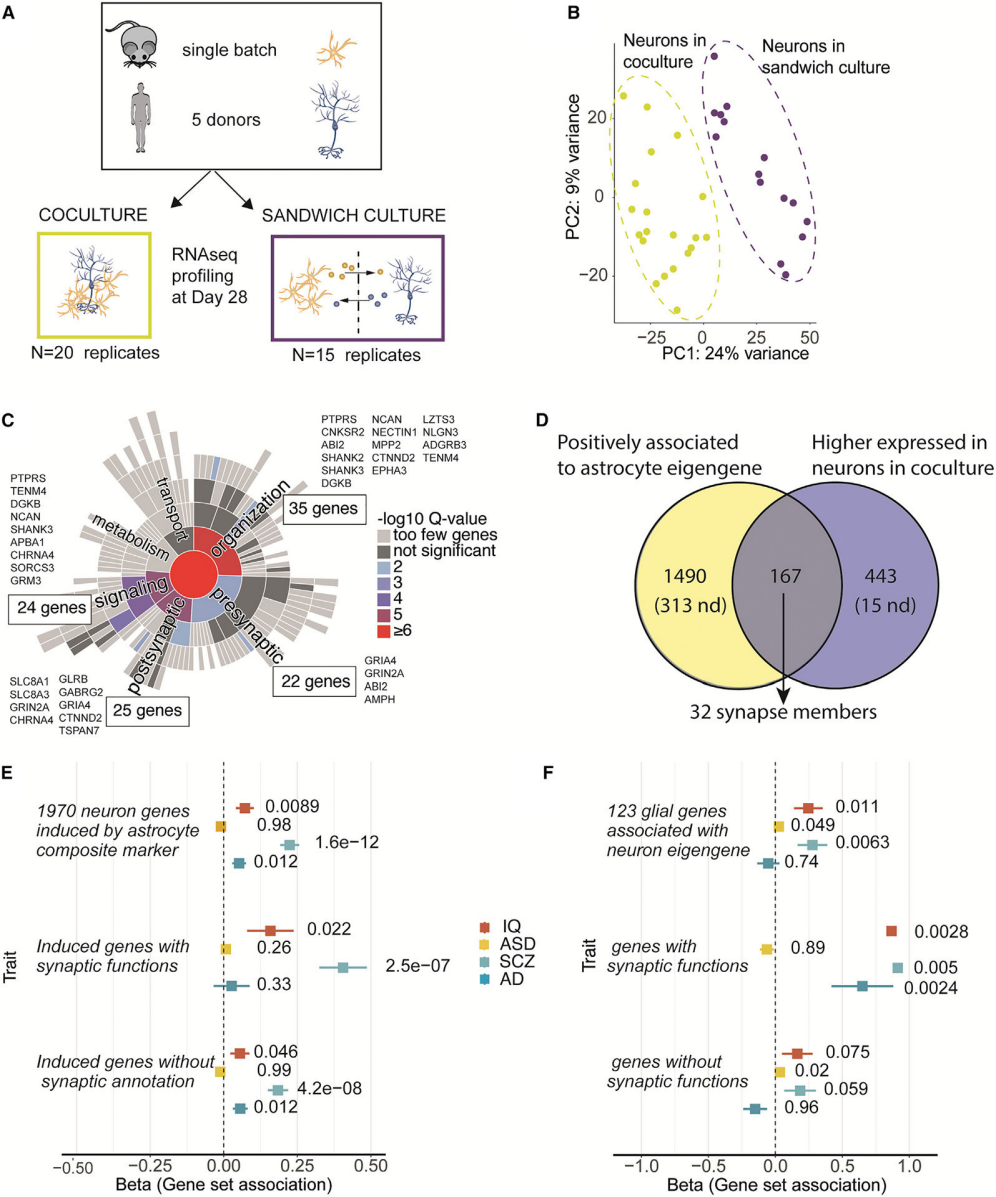
Synaptic programs in neurons are enhanced by physical contact with glial cells and involve genes that associate to schizophrenia (A) Experimental design for isogenic sandwich (n = 15) and coculture (n = 18) experiments. (B) PCA of transcriptome of neurons in coculture and sandwich culture (C) Physical contact with glial cells enhances expression of synaptic genes in neurons. Genes associated with the astrocyte eigengene are highlighted (n = 83). (D) Overlap of induced genes in coculture and genes with positive association to the astrocyte eigengene. 10% of genes induced in coculture are associated with high astrocyte eigengene values. (E and F) Gene set association analysis for four central nervous system traits in neurons (E) and glial cells (F). p values for gene set associations for each trait and gene set are indicated in the plot. Astrocytes induce transcripts encoded by genes associated with schizophrenia in neurons (E), and glia-expressed genes that promote neuronal maturation associate with schizophrenia (F). The error bars present the standard error (SE). Nd, not detected.

The association of neuronal-synaptic gene expression with astrocyte expression of synaptic cell adhesion molecules raised the question of whether physical contact between neurons and glial cells was required for the induction of the synaptic gene-expression programs in the neurons. To evaluate this, we carried out experiments (with five neuronal lines) in which neurons were differentiated either in a glia coculture or in a glia sandwich culture (Figure 7A). The sandwich culture enables the exchange of soluble factors in the culture media between the two cell types but prevents any physical contact. In contrast, in coculture, the cells are free to interact by both physical contact and exchanging soluble factors. We reasoned that differences in the transcriptome between neurons in the two culture conditions would indicate effects brought about by physical contact between glial cells and neurons. We generated bulk RNA-seq data at day 28 of the differentiation from five lines in the two conditions (4/3 replicates each, n = 35 samples). A PCA and hierarchical clustering of the transcriptomic data clustered the isogenic neuron lines by culture condition (coculture or sandwich culture) (Figures 7B and S5A). This suggested that physical contact between glial cells and neurons was able to induce reproducible changes in neuron state across multiple isogenic experiments.

We next performed a differential gene-expression analysis to identify genes whose expression levels were changed in neurons by physical contact with glial cells. The analysis revealed 1,691 genes that significantly (adjusted p < 0.05) differed in expression between neurons in coculture and in sandwich culture (Figure S5B; Table S14). Of these, 625 were more highly expressed in the cocultured neurons (adjusted p < 0.05). We then used GO term enrichment analysis to investigate whether specific biological processes would be differentially affected in the neurons in the two conditions. The 625 genes with higher expression in glia-cocultured neurons (with glial contact) were enriched for components of nervous system processes and functions in the synapse, with the strongest individual enrichment for chemical synaptic transmission (GO:0007268; n = 61 genes, FC = 2.7, q = 1.6 × 10^−9^; Table S15). A systematic analysis for curated synaptic components in the SynGO database confirmed 104 synaptic genes (q = 4.2 × 10^−11^), 83 with known roles in synapse processes (q = 1.7 × 10^−6^), in synaptic organization (n = 35, q = 1.9 × 10^−5^) and signaling (n = 24, q = 1.9 × 10^−5^) (Figure 7C; Table S16). This suggested that the physical contact between the two cell types on coculture enhanced synaptic gene programs in neurons beyond effects resulting from soluble factors.

We next asked whether the neuronal genetic programs that were associated with high astrocyte eigengene values could be in part driven by contact-dependent mechanisms with glial cells. A comparison of expression between isogenic neurons in sandwich culture and coculture revealed, on average, higher expression of the 1,970 genes (1,657 detected) that were associated with the astrocyte eigengene (p = 1.9 × 10^−26^, paired t test; Figures S5C and S5D). We next identified the overlapping set of genes between the 625 genes with higher expression in coculture than in sandwich culture and the 1,970 genes that were associated with astrocyte eigengene values. Intriguingly, we found 167 genes that had both high expression in cocultured neurons and were induced by high astrocyte eigengene values. This overlap was significantly larger (10% of detected genes that were positively associated with astrocyte eigengene, n = 1,657) than the total number of genes that had higher expression in cocultured neurons (4% of all genes that were detected in both datasets, 610/13,968; p = 1.1 × 10^−22^, binomial test; Figure 7D). This suggested that a set of the same genes that were associated with high astrocyte eigengene values were also induced in neurons with contact with glial cells. Of these genes, 32 were synaptic (Table S17), and they were particularly enriched for members in the post-synapse specialization (N = 19 genes, GO:0099572, q = 1.9 × 10^−8^) and included members in synapse organization, signaling, and postsynaptic processes (Figure 7C). Altogether, these findings suggested that the induction of neuronal genetic programs that associated with high values of the astrocyte eigengene were in substantial part driven by physical contact between the neurons and the glial cells. They further indicated that the contact-driven intercommunication between neurons and glia was particularly relevant in regulating expression of members in the postsynaptic components.

### Glial cells induce genetic programs related to schizophrenia in neurons

Given that recent genetic discoveries have highlighted neuronally expressed genes with functions in synaptic biology in severe psychiatric illnesses, including schizophrenia,^4,5,40,41^ we wondered whether these loci overlapped with those genes that were found to be induced in neurons by astrocytes. To address this question, we calculated gene-wise associations from gene-surrounding variants using summary statistics from a recent genome-wide association study (GWAS) for schizophrenia^5^ using MAGMA.^42^ Using the gene-wise associations, we performed a gene set analysis for the 1,970 genes that were induced by astrocytes in neurons. The analysis revealed a significant association for variants near the astrocyte-induced genes with schizophrenia (β = 0.22, p = 1.6 × 10^−12^) (Figure 7E; Table S18). We further divided the genes into those that possessed a synaptic annotation in SynGO^26^ and non-synaptic genes. This revealed that the association to schizophrenia was stronger, but not exclusive, to genes with known synaptic annotations induced by astrocytes (β_synaptic_ = 0.41, p = 2.5 × 10^−7^; β_non-synaptic_ = 0.18, p = 4.8 × 10^−8^). The induced expression of genes relevant to schizophrenia by astrocytes in neurons suggests that astrocytes regulate neuronal functions that go awry in schizophrenia.

To assess the specificity of the association with schizophrenia, we repeated the analysis for three other central nervous system phenotypes with variable ages of onset: autism spectrum disorder (ASD),^43^ general intelligence,^44^ and Alzheimer’s disease (AD).^45^ The 1,970 induced neuronal genes showed modest association for AD (β = 0.05, p = 0.012) and IQ (β = 0.07, p = 0.009), whereas there was no evidence for association with ASD (Figure 7E). As for schizophrenia, the association for intelligence was stronger for genes with synaptic annotations than without (β_synaptic_ = 0.16, p = 0.02; β_non-synaptic_ = 0.05, p = 0.05). To investigate whether the 1,970 induced neuronal genes were affected in patients with schizophrenia, we studied their overlap with genes that were previously identified as reduced in the prefrontal cortex of schizophrenia patients.^46^ Of 314 genes that had reduced expression in patients, 50 were among the 1,970 genes we found to be induced by astrocytes in neurons (1.35-fold enrichment, p = 0.028, binomial test). Together these results demonstrated that the presence of astrocytes in glial cultures was associated with dose-dependent induction of genetic programs relevant particularly for schizophrenia in the accompanying neurons, including synaptic function and neuronal maturation.

### Astrocytic genes that induce synaptic programs in neurons are associated with schizophrenia

The potential role of astrocyte-neuron interactions in schizophrenia prompted us to investigate whether the genes whose high expression in glial cells was linked to synaptic programs in neurons were involved in schizophrenia. We reasoned that because glial cells are central in regulating many of the neuronal processes that have previously been implicated by genetic studies in schizophrenia,^47^ disturbances in the glia-neuron regulatory interactions may also be relevant for the disease. A gene set analysis in MAGMA for the 123 glial genes whose high expression was linked to induction of synaptic programs found that these genes tended to harbor common variation that was associated with schizophrenia (β = 0.28, p = 0.0063) (Figure 7F; Table S19) and was strongly driven by the genes with known synaptic annotations in SynGO (β = 0.91, p = 0.0050).

To investigate individual genes underlying the gene set association for schizophrenia, we ranked the 123 glia genes according to the MAGMA *Z*-statistic of gene-wise associations (Figure S5E; Table S20). Among the genes with the strongest associations were synaptic genes, including the cell adhesion molecules *LRRC4, LRRC4B*, and *NRXN1*, as well as the astrocytic *LRP4* involved in regulation of glutamate transmission,^35^ and included genes in 10 genome-wide significant loci (*LRP4, AP3B2, LRRC4B, KCNB1, CCDC39, PCDHB4, MSI2, B9D1*, *NALCN*, and *SNX32*).^5^ In addition, two genes had direct genetic evidence from rare variant associations (*NRXN1* and *MAGI2*).^4^ Our results indicated that these genes’ expression in glial cells was associated with synaptic programs and neuronal induction of genes associated with schizophrenia. This further suggested that the neurobiological programs in neuron-glia interactions *in vitro* are relevant to schizophrenia biology.

## DISCUSSION

Here, we studied how glial cells impact neuronal cell state and maturation by analyzing the co-expression of genes between the two cell populations in coculture using cross-species-resolved RNA-seq data. Our analysis revealed that genetic programs in glial cells that covary in abundance with astrocyte markers induce pre- and postsynaptic programs in neurons that associate with schizophrenia (Figures 3 and 7). We discovered that the neuronal synaptic gene-expression programs were associated with high expression of astrocytic synaptic cell adhesion molecules, including neurexins (*Nrxn1*), neuroligins (*Nlgn1, Nlgn3*), and leucine-rich repeat transmembrane proteins (*Lrrc4, Lrrc4b*), with direct evidence from both common and rare variant associations to schizophrenia^4,5^ (Figures 5, 6, and 7). Our analysis revealed associations with additional synaptic genes expressed by astrocytes with functions related to synaptic vesicle cycle (Cadps, Nrxn1, Unc13B, and Prkcb), as well as glutamate receptor subunit Gria1 and synaptic potassium channels Kcnb1. Importantly, we found that the synaptic cell adhesion molecules were induced in astrocytes on coculture with neurons, suggesting that the synaptic-adhesion abilities of astrocytes are induced by the presence of neurons. Moreover, the glial cells in coculture had higher expression of genes encoding functions in the glutamate recycling pathway, implying that the cocultured astrocytes are recruited to participate in the neuronal processes of the glutamatergic neurons. Finally, we saw that the differentiating neurons in coculture enhanced gene programs characteristic of astrocyte maturation in the early post-natal murine astrocytes. This is also consistent with previous work supporting that neurons participate in the regulation of astrocytes.^16^

Genetic associations in schizophrenia are concentrated in genes that are highly expressed by neurons and cortical regions of the brain with roles in the synapse, transmission, and differentiation.^3-5^ This has focused much of the previous attention in the field on neurons. Besides neurons, astrocytes are active modulatory components of neural circuits that shape the structure and function of neuronal synapses and ultimately behavior through direct contacts and secreted factors.^48^ Indeed, together with the presynaptic and postsynaptic nerve terminals, the synaptically associated astrocytes compose a solid functional unit known as a tripartite synapse.^49^ Importantly, astrocytes express many gene products typically described as neuronal synaptic elements, including receptors for neurotransmitters, synaptic cell adhesion molecules, and components of synaptic-like vesicle cycle^29,30,34,50^ in which gene variants have been associated with risk for schizophrenia.^4,5,51^

We found that physical contact with astrocytes was required to induce neuronal synaptic gene expression (Figures 7A-7D). This is consistent with previous reports showing that contact between astrocytes and neurons is required for synapse formation.^29,52^ Transsynaptic cell adhesion molecules, such as neurexins (NRXN), neuroligins (NLGN), and leucine-rich repeat transmembrane protein (LRRTM), provide a structural scaffold that holds synaptic terminals together and participate from early steps of synapse formation to regulating synaptic plasticity.^53,54^ Astrocytes were recently shown to express *NRXN1* and *NLGN1*, implying that they could also be involved in fastening astrocyte processes to the synapse.^29,30^ Importantly, deletion of *NRXN1* in astrocytes has been reported to affect synapse function by impairing the maturation of silent synapses, AMPA-receptor recruitment, and long-term potentiation without affecting the number of synapses.^30^ Postsynaptic LRRC4 and LRRC4B associate with postsynaptic density (PSD95) to regulate excitatory synapse formation by LRRC4 binding to presynaptic netrin-G2 (NTNG2) and LRRC4B binding to receptor tyrosine phosphatases LAR (PTPRF), PTPRS, and PTPRD to induce functional presynaptic differentiation in contacting neurites.^55-57^ In a manner similar to transsynaptic binding of NRXN1 and NLGN1,^58^ the heterophilic connections with LRRC4 and LRRC4B to their binding partners enable correct connectivity between presynaptic and postsynaptic terminals.^55,57^ Here, we report that expression of these genes encoding for synaptic cell adhesion molecules in glial cells correlates with the expression of genes with roles in synaptic organization, including structural transsynaptic adhesion molecules in neurons (Figure 3). Importantly, we found that *NTNG2* and *PTPRS* that bind LRRC4 and LRRC4B, respectively, were induced in neurons in response to astrocytes. These findings provide further evidence that these molecules participate in glia-neuron interactions that may involve synaptic contacts. The expression of heterophilic synaptic cell adhesion molecules characteristic for presynaptic and postsynaptic nerve terminals in the glial cells suggests that they may be involved in positioning glial processes with connections to the two neuronal synaptic terminals.

Astrocytes are central in regulating many of the key neuronal functions, related to development, synaptogenesis, maturation, and synaptic transmission that are fundamental to all information processing in the brain.^7^ Together with the discoveries of synaptic gene variants in schizophrenia, there has been increasing interest in astrocytes’ potential role in the disorder.^47,59^ Complement proteins are expressed by neurons and glial cells, including astrocytes, and are localized to a subset of developing synapses.^60,61^ Defects in developmental pruning can lead to synaptic loss long before onset of clinical symptoms in line with the developmental model. Here we show that early developmental glia interactions with hPSC-derived neurons with resemblance to late prenatal stages^12^ are associated with coregulated expression of synaptic adhesion genes linked with schizophrenia.

Follow-up studies of recent genetic discoveries comparing gene-expression patterns across tissues and cell types have implicated neurons as the cell type in which schizophrenia-linked genes tend to have the strongest expression on average.^3-5^ However, many emblematic neuronal functions depend on the interplay with glial cells and may impact the pathology through direct or cell-nonautonomous effects on neurons. Here we show that expression of schizophrenia-associated genes in glial cells is correlated with a neuronal maturation state and induction of the expression of risk genes in neurons. Our results underscore the importance of evaluating the converging functional impact of emerging genetic discoveries in living biological model systems that provide mechanistic insight, such as cellular interactions, into affected biological processes.

### Limitations of the study

Although we show here that synaptic gene programs in neurons are correlated with the expression of pro-synaptic genes in glia, it remains unknown how these interactions are mediated. We have conducted a series of experiments in mixed cultures of human induced neurons and mouse glial cells that revealed a potential relevant cellular context for schizophrenia. While mouse glial cells have been shown to enhance neuronal maturation, it is possible that they lack abilities that are inherent for human cell types and will be present only in fully human coculture systems. As human astrocyte and human neuron coculture paradigms are developed and optimized, it will be important to test to which extent the cellular programs we report here are conserved. Overall, the *in vitro* cell cultures are a powerful experimental platform to study cellular responses to their surroundings. However, this approach does not fully recapitulate the complexity of the *in vivo* cellular context in the brain. Instead, it aims to illuminate individual components of neuronal interactions with other cell types. Finally, schizophrenia is a behavioral human-specific phenotype with both genetic and environmental influences.^62,63^ It is, therefore, important to note that we do not model schizophrenia but cellular processes underlying the disorder.

## STAR★METHODS

### RESOURCE AVAILABILITY

#### Lead contact

Further information and requests for resources and reagents should be directed to and will be fulfilled by the lead contact, Ralda Nehme (rnehme@broadinstitute.org).

#### Materials availability

This study did not generate new unique reagents.

#### Data and code availability

The raw data reported in this study cannot be deposited in a public repository due to varied consent provenance within our selected cohort and subsequent data access restrictions. In addition, summary statistics describing these data are available in Tables S1-S21. Subsets of the data will be made available by the corresponding authors upon reasonable request within a 30-day time frame under a data transfer agreement. This paper does not report original code. Any additional information required to reanalyze the data reported in this paper is available from the lead contact upon request.

### EXPERIMENTAL MODEL AND SUBJECT DETAILS

#### Human pluripotent stem cell lines

29 previously described hiPSC lines were used in this study^18^ (Table S1). In addition, 51 human embryonic stem cell (hESC) lines were used, also previously described.^27,72^ All hiPSC and hESC lines are listed in Table S1 and S6 and the key resources table.

### METHOD DETAILS

#### hPSC culture

Human ESCs and iPSCs were maintained on plates coated with geltrex (life technologies, A1413301) in StemFlex medium (Gibco^™^, A3349401) supplemented with Normocin^™^ Antimicrobial Reagent (Invivogen, Ant-nr-1). and passaged with accutase (Gibco, A11105). All cell cultures were maintained at 37°C, 5% CO2.

#### Infection of hPSCs with lentiviruses

Lentivirus particles were produced by Alstem (http://www.alstembio.com/). hPSCs were seeded in a geltrex-coated 12 well plate at a density of 100,000 cells/cm^2^ in StemFlex medium supplemented with ROCK-Inhibitor (Y27632, Stemgent 04-0012) and lentiviruses containing a murine Neurogenin 2 (Ngn2) tagged with a puromycin resistance gene and a tetracycline inducible GFP, at a MOI (multiplicity of infection) of 2. Cells were plated at a density of 100,000 cells/cm2 and incubated while in suspension with media containing 10 μM ROCK-Inhibitor (Sigma, Y27632). After 24 h, the medium was changed to StemFlex. The cells were grown until confluency, and then either maintained as stem cells, passaged, banked, or induced with Doxycycline for neuronal differentiation.

#### Neuronal differentiation

Neuronal differentiation of PSCs into cortical glutamatergic neurons was carried out as previously described.^12^ In brief, the differentiation was carried out by adding Doxycycline hyclate (2 μg/mL) to N2 supplemented media (Thermo Fisher, 17502048,) with patterning factors SB431542 (Tocris, 1614, 10 μM), XAV939 (Stemgent, 04-00046, 2 μM) and LDN-193189 (Stemgent, 04-0074, 100 nM), as described previously.^12,73,74^ Puromycin selection was used (5 μg/μL), from days 2–6 to remove non-transduced cells. At 4 days post induction, neuronal cells were resuspended into Neurobasal media (Gibco, 21103049) that was supplemented with B27 (Gibco, 17504044, 50X), doxycycline (2 μg/mL), brain-derived neurotrophic factor (BDNF), ciliary neurotrophic factor (CTNF), and glial cell-derived neurotrophic factor (GDNF) (R&D Systems 248-BD/CF, 257-33 NT/CF, and 212-GD/CF at 10 ng/mL each). From this point onwards the neurons were either co-cultured with murine glial cells that were derived from early postnatal (P1-P3) mouse brains as described previously^19^ or were left to grow as monocultures (mouse strain https://www.jax.org/strain/100012; animal ethical committee approval by Harvard University: Animal Experimentation Protocol (AEP) # 93-15). To evaluate whether physical contact between neurons and glia was required for some of the transcriptional effects, we used a sandwich culture setup whereby glia cells were cultured at the bottom as a monolayer, and neuronal cells were grown on a membrane insert (Sigma, CLS3450).

#### Cell villages

The cell villages were generated as described previously.^27,28^ At day 3 of the differentiation, the differentiating cells from 48 donors were passaged into differentiation media that was supplemented with 5-Ethynyl-2’-deoxyuridine (Life Technologies, A10044, 10 μM). Cell villages of the immature neurons were generated at day 6 by dissociating the cells with Accutase^®^, counting them by the Scepter^™^ Automated Cell Counter (Millipore Sigma) and plating the cells at a density of 40 000 cells/cm2 in Neurobasal media (Gibco, 21103049) supplemented with B27 (Gibco, 17504044, 50X), doxycycline (2 μg/mL), brain-derived neurotrophic factor (BDNF), ciliary neurotrophic factor (CTNF), and glial cell-derived neurotrophic factor (GDNF) (R&D Systems 248-BD/CF, 257-33 NT/CF, and 212-GD/CF at 10 ng/mL each). Neuronal villages were either grown as monocultures or co-cultured with murine glial cells (at a density of 70 000 cells/cm2). Villages were harvested for single cell RNA sequencing at day 28 of the differentiation.

#### Immunohistochemistry

Cultures neurons were fixed at D28 of neuronal differentiation in 4% paraformaldehyde +5% sucrose in DPBS for 20 min at room temperature. Cells were incubated with blocking buffer containing 4% horse serum, 0.1M Glycine, and 0.3% Triton-X in PBS for 1 h at room temperature. Primary antibodies, diluted in 4% horse serum in PBS, were incubated overnight at 4°C. Secondary antibodies were diluted in 4% horse serum in PBS and applied for 1 h at room temperature. Samples were washed 3x with PBS and imaged on spinning disc confocal microscope (Andor Dragonfly) with a 20x air interface objective using Fusion software. The following antibodies were used: chicken anti-MAP2 (1:10,000, Abcam ab5392), Rabbit anti-Syn-1 (1:1000 millipore AB1543P), Rabbit anti-GFAP (1:250, Abcam ab16997), Rabbit anti-Cx43 (1:500, ab230537). Goat Alexafluor plus-555- conjugated anti-mouse (Cat # A32727) and Goat anti-Rabbit Alexafluor plus-488 (Cat # A32731).

#### RNA sequencing

For bulk RNA sequencing cells were harvested in RTLplus Lysis buffer (Qiagen 1053393) and stored at −80°C. Each experiment was conducted in 3-4 replicates to reduce experimental variability. Sequencing libraries were generated from 100 ng of total RNA using the TruSeq RNA Sample Preparation kit (Illumina RS-122-2303) and quantified using the Qubit fluorometer (Life Technologies) following the manufacturer’s instructions. Libraries were then pooled and sequenced by high output run on a HiSeq 2500 (Illumina). Cells grown using the sandwich culture set up (along with neuron monocultures from the same cell lines and glia monocultures from the same batch) were processed and sequenced at the Broad Genomics Platform using the Smartseq2 workflow.^75^ The RNA-seq fastq data was aligned to ENSEMBL human reference genome (build GRCh37.p13/hg19) and mixed human-mouse reference genome (GRCh37/hg19 and GRCm38/mm10, GSE63269)^22^ by STAR (v.2.5).^64^ Prior to genome aligning, the Illumina adapters and low-quality base-pairs were clipped from the ends of the sequence reads by Trimmomatic (v.0.36)^65^ and reads with length <36 base-pairs were removed. Reads were aligned and annotated using GENCODE GTF annotation version 19 and a custom GTF file for the mixed genome (GSE63 2 69)^22^ for the mixed genome. The aligned reads were then quantified into a counts matrix using the featureCounts method from Rsubread (v.1.32).^66^ The read counts from the three experimental replicates were summed together yielding an average library size of (11 × 10^6^ reads). The final analyses were carried out from these summed read counts of genes from the mixed alignment reference.

Genotype data was previously generated from all donors in the village and processed to a joint VCF.^27^ This VCF was then made to have only biallelic variants, then filtered to variants which pass VQSR, have a combined read depth >10 across samples, have <1% chance of being incorrectly called, and are present in more than half the donors included in the VCF, have a probability of deviating from Hardy-Weinberg Equilibrium <0.001, and that have a minor allele frequency of 0.1 or greater.

For single cell RNA sequencing of neuron villages, the cells were harvested, and RNA samples prepared with 10X Chromium Single Cell 3′ Reagents V3 followed by sequencing with Illumina NovaSeq 6000 (Illumina) using an S2 flow cell at 2 × 100bp, as previously described.^28,72^ Raw sequence fastq-files were then demultiplexed and aligned according to the Drop-seq workflow^22^ to the mixed human and mouse reference genome (GRCh38, and GRCm38/mm10, *annotated with* ensembl v89 gene model), removing any reads originating from murine cells, and filtered for high quality mapped reads (MQ > 10). Donor reference genotype data was processed as previously described and aligned to human reference (GRCh38).^27^ The Dropulation single-donor assignment algorithm was then run on the preprocessed sequence data and the donor genotypes VCF to accurately specify the donor identity of each droplet, as specified previously.^28^ The Dropualtion algorithm analyzes each cell independently and generates a probability of the data being generated from each of the donors in the VCF file. These probabilities are used to identify and remove cell barcodes assigned to more than one cell (doublets) and cell barcodes with no confidently assigned donors are also removed. Remaining cells are then assigned to the donor with the highest likelihood of generating them.^28^ A cell by genes matrix (digital expression matrix) was generated for each library, and the UMI counts for all cells per donor were summed to generate a donor by gene matrix (meta-cell matrix). Differential gene expression analysis was performed on the meta-cell matrices with limma/voom while adjusting for covariates.

### QUANTIFICATION AND STATISTICAL ANALYSIS

#### Differential gene expression

Differential expression analysis of bulk RNAseq data was performed on neurons grown with and without glia on day 28 of differentiation using the limma-voom (v. 3.34.9) package.^25^ Estimated read counts were normalized for library size (method = TMM) and by voom to produce log_2_-transformed normalized gene expression estimates. Genes with low read counts (≤ 10 reads in at least one library and total read count ≤15) were removed by filterByExpr function in edgeR (v. 3.20.9) prior to normalization. For differential expression analysis of the astrocyte eigengene effect on neuronal gene expression a multifactorial model was used with astrocyte eigengene and normalized Ngn2 expression as covariates (~ AstroE + ngn2). The proportion of variance explained by each covariate was estimated with variancePartition package (v.1.8.1). For differential expression analysis of mouse RNAseq data a linear model with neuron eigengene as a covariate was used (~NeuroE). The p values were adjusted with FDR <5% and Bonferroni correction for transcriptome-wide significance. PCA was performed on data from day 4 NPCs together with day 4 neurons – ComBat (SVA v. 3.26.0) was used to correct for experimental batches and counts were normalized to log_2_ counts per million prior to calculating PCA. For analyzing the effect of physical contact between glial cells and neurons, we used bulk RNAseq data from isogenic experiments of neurons differentiated in glia coculture or in sandwich. For differential expression analysis of the human reads, variancePartition was used to adjust for donor effect, as well as for sequence library quality measures from Picard tools (PCT_USABLE_BASES and MEDIAN_CV_COVERAGE), which were found to differ significantly between the isogenic experiments. The impact of neuron presence on glial cells was estimated by joint differential expression analysis of mouse genes from 4 replicates of glia alone cultures and 5 glia-neuron cocultures. The experimental replicates (4 from each donor) from the cocultured glial cells were summed together to match the glia library size of the four replicates from glia alone cultures. The raw reads were then filtered (>10 reads in at least one library and total read count >15) and normalized by TMM-method in limma-voom package. Differential expression analysis was done between the two culture conditions using the formula (~0 + culture.condtion). For analyzing the effect of glia coculture on neurons, UMIs of all donor cells in the two culture conditions were summed to generate meta cells. The differential expression analysis weas carried out for human genes in limma-voom between neurons grown with or without glial cells. The analysis was adjusted for donor sex, average number of UMIs per cell and number of cells of each donor as covariates. The donor effect was adjusted for using the block design in limma.

#### Marker gene expression and eigengene generation

Expression of canonical marker genes from previous literature were used to characterize neuron and glial cell populations in the different experiments (Table S2). Singular value decomposition was used to generate astrocyte and neuron eigengenes from log_2_ normalized read counts per million (CPMs) using svd function in R. An m × n matrix of genes (m) and cell lines (n) for six canonical astrocyte marker genes selected from literature were used to generate the astrocyte eigengene (the first right singular vector v). The average variance explained by the eigengene was calculated from the squared singular value (d) of each gene divided by their sum $\frac{d^{2}}{\sum d^{2}}$. The astrocyte eigengene values for the joint analysis of the discovery and deletion lines was calculated jointly. The eigengene value for glia-free cultures were set to zero. For neuron eigengene, 1,970 genes that were found positively associated with astrocytes were used to calculate the first singular vector (v).

#### Gene set enrichment analysis

GO term enrichment was analyzed using enrichGO function in clusterProfiler package (v. 3.8.1) in R with org.Hs.eg.db and org. Mm.eg.db (v. 3.6.0.) for human and mouse genes, respectively, using ENSEMBL identifiers. A minimum of 10 and maximum of 1000 genes per category were included to the analysis. p-values for adjusted for multiple testing by Benjamini & Hochberg method with p value <0.01 and q-value <0.05 cutoffs. A custom gene universe was used as the background for the enrichment analysis including only genes that were analyzed in the RNAseq data. The GO terms for each category were analyzed separately. For synaptic gene annotations we used manually curated annotations from the SynGO database.^26^ Enrichment analysis with Synaptic GO terms were analyzed in the SynGO online portal (v.1.0) using custom gene universe of genes that were included in RNAseq data analysis (www.syngoportal.org).

#### Mouse brain single cell atlas

A single cell gene expression atlas from adult mouse brain was used to analyze the expression of glial genes *in vivo*.^31^ Meta cells for available non-neuronal cell types including polydendrocytes, microglia, astrocyte, oligodendrocyte and macrophacge were down-loaded and processed in limma-voom. Astrocyte meta cell was formed as the average of Gja1 positive and Gja1/Myoc positive meta cells. The gene expression in the glial meta cells was standardized to *Z* score values and t test was used to analyze differences in average expression between the meta cells.

#### Gene set association analysis

Summary statistics from recent well-powered GWAS studies for four central nervous system traits, including schizophrenia,^5^ ASD,^43^ measure of general intelligence,^44^ and AD^45^ were used to calculate gene-wise association in MAGMA (v.1.07b).^42^ SNPs from GWAS summary statistics were annotated to genes using 10kb window (window = 10) and gene locations from human genome build GRCh37.3 provided on the software website. The gene-wise associations were then calculated based on SNP annotations using 1000 genomes references data of European ancestry^76^ for estimating the LD structure. Gene-set associations were analyzed from gene-wise associations with –gene-set flag for specified gene sets from the differential expression analysis. For mouse genes, human orthologues were identified prior to the analysis with getLDS function in biomaRt package (v. 2.34.2) using BioMart ENSEMBL databases for mouse and human.

## Supplementary Material

Supp file 4

Supp file 3

Supp file 12

Supp file 6

Supp file 7

Supp file 11

Supp file 5

Supp file 10

Supp file 15

Supp file 14

Supp file 13

Supp file 2

Supp file 1

Supp file 9

Supp file 16

Supp file 8

Supp file 17

Supp file 18

## Figures and Tables

**Table:** KEY RESOURCES TABLE

REAGENT or RESOURCE	SOURCE	IDENTIFIER
Antibodies		
chicken anti-MAP2	Abcam	Cat#ab5392; RRID:AB_2138153
Rabbit anti-Syn-1	Millipore	Cat#AB1543P; RRID:AB_90757
Rabbit anti-GFAP	Abcam	Cat#ab16997; RRID:AB_443592
Rabbit anti-Cx43	Abcam	Cat#ab230537
Goat Alexafluor plus-555-conjugated anti-mouse	Thermofisher	Cat#A32727; RRID:AB_2633276
Goat anti-Rabbit Alexafluor plus-488	Thermofisher	Cat#A32731; RRID:AB_2633280
Experimental models: Cell lines		
HUES53 hESC line	Eggan lab	HUES53
Hes3 hESC line	Eggan lab	Hes3
HUES62 hESC line	Eggan lab	HUES62
HUES63 hESC line	Eggan lab	HUES63
SCBB-1438 hiPSC line	Stanley Center Stem Cell Resource	SCBB-1438
SCBB-1471 hiPSC line	Stanley Center Stem Cell Resource	SCBB-1471
SCBB-1472 hiPSC line	Stanley Center Stem Cell Resource	SCBB-1472
SCBB-1473 hiPSC line	Stanley Center Stem Cell Resource	SCBB-1473
SCBB-1477 hiPSC line	Stanley Center Stem Cell Resource	SCBB-1477
SCBB-1478 hiPSC line	Stanley Center Stem Cell Resource	SCBB-1478
SCBB-1479 hiPSC line	Stanley Center Stem Cell Resource	SCBB-1479
SCBB-1480 hiPSC line	Stanley Center Stem Cell Resource	SCBB-1480
SCBB-1481 hiPSC line	Stanley Center Stem Cell Resource	SCBB-1481
SCBB-1483 hiPSC line	Stanley Center Stem Cell Resource	SCBB-1483
SCBB-1489 hiPSC line	Stanley Center Stem Cell Resource	SCBB-1489
SCBB-1645 hiPSC line	Stanley Center Stem Cell Resource	SCBB-1645
SCBB-1646 hiPSC line	Stanley Center Stem Cell Resource	SCBB-1646
SCBB-1647 hiPSC line	Stanley Center Stem Cell Resource	SCBB-1647
SCBB-1648 hiPSC line	Stanley Center Stem Cell Resource	SCBB-1648
SCBB-1827 hiPSC line	Stanley Center Stem Cell Resource	SCBB-1827
SCBB-1828 hiPSC line	Stanley Center Stem Cell Resource	SCBB-1828
SCBB-228 hiPSC line	Stanley Center Stem Cell Resource	SCBB-228
SCBB-229	Stanley Center Stem Cell Resource	SCBB-229
SCBB-243	Stanley Center Stem Cell Resource	SCBB-243
SCBB-258	Stanley Center Stem Cell Resource	SCBB-258
SCBB-269	Stanley Center Stem Cell Resource	SCBB-269
SCBB-652	Stanley Center Stem Cell Resource	SCBB-652
SCBB-653	Stanley Center Stem Cell Resource	SCBB-653
SCBB-798	Stanley Center Stem Cell Resource	SCBB-798
SCBB-799	Stanley Center Stem Cell Resource	SCBB-799
SCBB-800	Stanley Center Stem Cell Resource	SCBB-800
SCBB-803	Stanley Center Stem Cell Resource	SCBB-803
CHB5_P25_140801 hESC line	Eggan lab	CHB5
CHB9_P24_140728 hESC line	Eggan lab	CHB9
CSES12_P14_140616 hESC line	Eggan lab	CSES12
CT2_P11_140622 hESC line	Eggan lab	CT2
CT4_P15_140622 hESC line	Eggan lab	CT4_
ESI049_P33_140612 hESC line	Eggan lab	ESI049
Genea15_P20_150110 hESC line	Eggan lab	Genea
Genea16_P17_150104 hESC line	Eggan lab	Genea
Genea42_P19_150107 hESC line	Eggan lab	Genea
Genea43_P12_150104 hESC line	Eggan lab	Genea
Genea47_P10_150104 hESC line	Eggan lab	Genea
Genea52_P11_150110 hESC line	Eggan lab	Genea
Genea57_P13_150110 hESC line	Eggan lab	Genea
HS346_P25_140704 hESC line	Eggan lab	HS346
HS401_P25_140619 hESC line	Eggan lab	HS401
HS420_P24_140623 hESC line	Eggan lab	HS420
HUES42_P22_131008 hESC line	Eggan lab	HUES42
HUES44_P16_131008 hESC line	Eggan lab	HUES44
HUES45_P21_131206 hESC line	Eggan lab	HUES45
HUES62_P18_131007 hESC line	Eggan lab	HUES62
HUES63_P15_140416 hESC line	Eggan lab	HUES63
HUES72_P20_150119 hESC line	Eggan lab	HUES72
HUES74_P7_150201 hESC line	Eggan lab	HUES74
HUES75_P7_150124 hESC line	Eggan lab	HUES75
Mel1_P20_150107 hESC line	Eggan lab	Mel1
Mel4_P37_150119 hESC line	Eggan lab	Mel4
RUES1_P25_140730 hESC line	Eggan lab	RUES1
UCLA10_P15_150817 hESC line	Eggan lab	UCLA10
UCLA13_P16_150817 hESC line	Eggan lab	UCLA13
UCLA14_P20_150817 hESC line	Eggan lab	UCLA14
UCLA16_P16_150817 hESC line	Eggan lab	UCLA16
UCLA1_P15_150817 hESC line	Eggan lab	UCLA1
UCLA2_P14_150817 hESC line	Eggan lab	UCLA2
UCLA8_P15_150817 hESC line	Eggan lab	UCLA8
UCSF4_P19_140613 hESC line	Eggan lab	UCSF4
UM14.1_P26_150115 hESC line	Eggan lab	UM14.1
UM33.4_P25_150107 hESC line	Eggan lab	UM33.4
UM4.6_P15_150116 hESC line	Eggan lab	UM4.6
UM77.2_P12_150107 hESC line	Eggan lab	UM77.2
WA01_P23_140528 hESC line	Eggan lab	WA01
WA14_P21_131206 hESC line	Eggan lab	WA14
WA17_P12_140530 hESC line	Eggan lab	WA17
WA18_P9_140530 hESC line	Eggan lab	WA18
WA22_P14_140530 hESC line	Eggan lab	WA22
WA7_P33_140529 hESC line	Eggan lab	WA7
WIBR3_P23_140624 hESC line	Eggan lab	WIBR3
WIBR5_P13_140611 hESC line	Eggan lab	WIBR5
WIBR6_P11_140617 hESC line	Eggan lab	WIBR6
Software and algorithms		
STAR (v.2.5)	Dobin et al., 2013^64^	GitHub alexdobin/STAR
Trimmomatic (v.0.36)	Bolger et al., 2014^65^	Usadellab.org
Rsubread (v.1.32)	Liao et al., 2014^66^	bioconductor.org/
Drop-seq workflow	Macosko et al., 2015^22^	mccarrolllab.org/dropseq/
edgeR (v. 3.20.9)	Robinson et al., 2010^67^	bioconductor.org/
limma-voom (v. 3.34.9)	Law et al., 2014^25^	bioconductor.org/
variancePartition (v.1.8.1)	Hoffman, and Schadt, 2016^68^	bioconductor.org/
SVA (v. 3.26.0)	Leek et al., 2012^69^	bioconductor.org/
clusterProfiler (v. 3.8.1)	Yu et al., 2012^70^	bioconductor.org/
SynGO online portal (v.1.0)	Koopmans et al., 2019^26^	syngoportal.org
MAGMA (v.1.07b)	de Leeuw et al., 2015^42^	bioconductor.org/
biomaRt (v. 2.34.2)	Durinck et al., 2005^71^	bioconductor.org/

## Highlights

- Major gene-expression changes occur in neurons and glia on contact
- Synaptic gene-expression programs are enhanced in neurons
- Expression of genes that encode synaptic cell adhesion molecules is increased in glia
- Both programs are enriched for genes associated with schizophrenia risk
