# Supplementary material for: Astrocytic cell adhesion genes linked to schizophrenia correlate with synaptic programs in neurons
Source: Cell Rep. Author manuscript; Available in PMC 2023 Dec 14. (PMC10721115; doi:10.1016/j.celrep.2022.111988)
Supplement: Supp file 3 [file NIHMS1946831-supplement-Supp_file_3.pdf]

Cell Reports, Volume *42*

Supplemental information

Astrocytic cell adhesion genes linked to schizophrenia correlate with synaptic programs in neurons

Olli Pietiläinen, Aditi Trehan, Daniel Meyer, Jana Mitchell, Matthew Tegtmeyer, Vera Valakh, Hilena Gebre, Theresa Chen, Emilia Vartiainen, Samouil L. Farhi, Kevin Eggan, Steven A. McCarroll, and Ralda Nehme

Table S2. Canonical marker genes for cell type identities. Related to Figure 3.

Gene name	Marker identity	Species
POU5F1/Oct4	stem cell	human
MKI67	stem cell	human
DCX	neural identity	human
MAP2	neural identity	human
MAPT	neural identity	human
NCAM1	neural identity	human
RBFOX3	neural identity	human
SYN1	neural identity	human
TUBB3	neural identity	human
EMX2	neuronal progenitor	human
HES1	neuronal progenitor	human
MSI1	neuronal progenitor	human
NEUROD1	neuronal progenitor	human
OTX2	neuronal progenitor	human
Aldh1l1	astrocyte	mouse
Slc1a3	astrocyte	mouse
Slc1a2	astrocyte	mouse
Gfap	astrocyte	mouse
Notch1	astrocyte	mouse
S100b	astrocyte	mouse
Cnp	oligodendrocytes	mouse
Mag	oligodendrocytes	mouse
Mog	oligodendrocytes	mouse
Mbp	oligodendrocytes	mouse
Cspg4	oligodendrocytes	mouse
Olig2	oligodendrocytes	mouse
Sox10	oligodendrocytes	mouse
Pycard	microglia	mouse
Itgam	microglia	mouse
Cd40	microglia	mouse
Ptprc	microglia	mouse
Cd68	microglia	mouse
Adgre1	microglia	mouse
Alf1	microglia	mouse
Spi1	microglia	mouse
Tmem119	microglia	mouse
C1qa	microglia homeostatic	mouse
C1qb	microglia homeostatic	mouse
Cx3cr1	microglia homeostatic	mouse
Cst3	microglia homeostatic	mouse

P2ry12	microglia homeostatic	mouse

Table S18. Gene set association results for CNS traits for genes induced in neurons by astrocytes. Related to Figure 7.

NGENES	BETA	BETA_STD	SE	P	Trait	Cell type	Neuron gene set
1603	0.07168	0.01974	0.03025	0.0089136	Intelligence	neurons	All induced genes (FDR < 5%)
1529	0.05221	0.01427	0.02324	0.012329	AD	neurons	All induced genes (FDR < 5%)
1547	-0.00961	-0.00268	0.00444	0.98473	ASD	neurons	All induced genes (FDR < 5%)
1565	0.22387	0.06228	0.03209	1.57E-12	schizophrenia	neurons	All induced genes (FDR < 5%)
219	0.15833	0.01673	0.07892	0.022417	Intelligence	neurons	Induced synaptic genes (FDR < 5%)
208	0.02687	0.00281	0.06090	0.3295	AD	neurons	Induced synaptic genes (FDR < 5%)
212	0.00732	0.00078	0.01130	0.25858	ASD	neurons	Induced synaptic genes (FDR < 5%)
217	0.40510	0.04360	0.08055	2.49E-07	schizophrenia	neurons	Induced synaptic genes (FDR < 5%)
1383	0.05463	0.01406	0.03242	0.045981	Intelligence	neurons	Induced non-synaptic genes (FDR < 5%)
1320	0.05580	0.01425	0.02487	0.01244	AD	neurons	Induced non-synaptic genes (FDR < 5%)
1334	-0.01211	-0.00315	0.00473	0.99476	ASD	neurons	Induced non-synaptic genes (FDR < 5%)
1347	0.18399	0.04779	0.03432	4.21E-08	schizophrenia	neurons	Induced non-synaptic genes (FDR < 5%)

Table S19. Results for gene set associations to CNS traits in glial cells. Related to Figure 7.

NGENES	BETA	BETA_STD	SE	P	Trait	cell type	Glial gene set
117	0.24610	0.01906	0.10666	0.01053	Intelligence	glial cells	Genes positively associated to neuron eigengene
113	-0.05260	-0.00406	0.08267	0.73769	AD	glial cells	Genes positively associated to neuron eigengene
115	0.02617	0.00207	0.01581	0.04898	ASD	glial cells	Genes positively associated to neuron eigengene
116	0.27640	0.02181	0.11079	0.00631	schizophrenia	glial cells	Genes positively associated to neuron eigengene
15	0.86757	0.02412	0.31360	0.00284	Intelligence	glial cells	Synaptic genes positively associated to neuron eigengene
13	0.64955	0.01707	0.23029	0.00240	AD	glial cells	Synaptic genes positively associated to neuron eigengene
13	-0.06292	-0.00168	0.05209	0.88646	ASD	glial cells	Synaptic genes positively associated to neuron eigengene
14	0.91359	0.02512	0.35489	0.00503	schizophrenia	glial cells	Synaptic genes positively associated to neuron eigengene
101	0.16398	0.01180	0.11385	0.07490	Intelligence	glial cells	Non-synaptic genes positively associated to neuron eigengene
99	-0.15064	-0.01090	0.08868	0.95531	AD	glial cells	Non-synaptic genes positively associated to neuron eigengene
101	0.03426	0.00254	0.01675	0.02043	ASD	glial cells	Non-synaptic genes positively associated to neuron eigengene
101	0.18440	0.01358	0.11766	0.05855	schizophrenia	glial cells	Non-synaptic genes positively associated to neuron eigengene

Figure S1. Alignment effect on transcriptome. A) Representative images of neuron monocultures and neuron-glia cocultures from 4 independent cell lines, staining for left: DAPI (blue) GFAP (green) and MAP2 (magenta), and right: DAPI (blue) CX43 (red) Syn1 (green) and MAP2 (magenta), Scale bar= 100um. B) Variance partitioning by human and mixed reference genome reveals 329 genes for which cross-species mapping explains >10% of variation in read counts. On average cross-species mapping explains only 0.78% of the variation in the read counts. C) PCA components 7 and 8 the cluster data set according to alignment. D) PCA of RNAseq data from day 28 neurons aligned to mixed reference genome. E) Canonical marker gene expression for pluripotency, neuronal progenitor cells and neuronal identity at different stages of differentiation and culture conditions. Related to Figure 2.

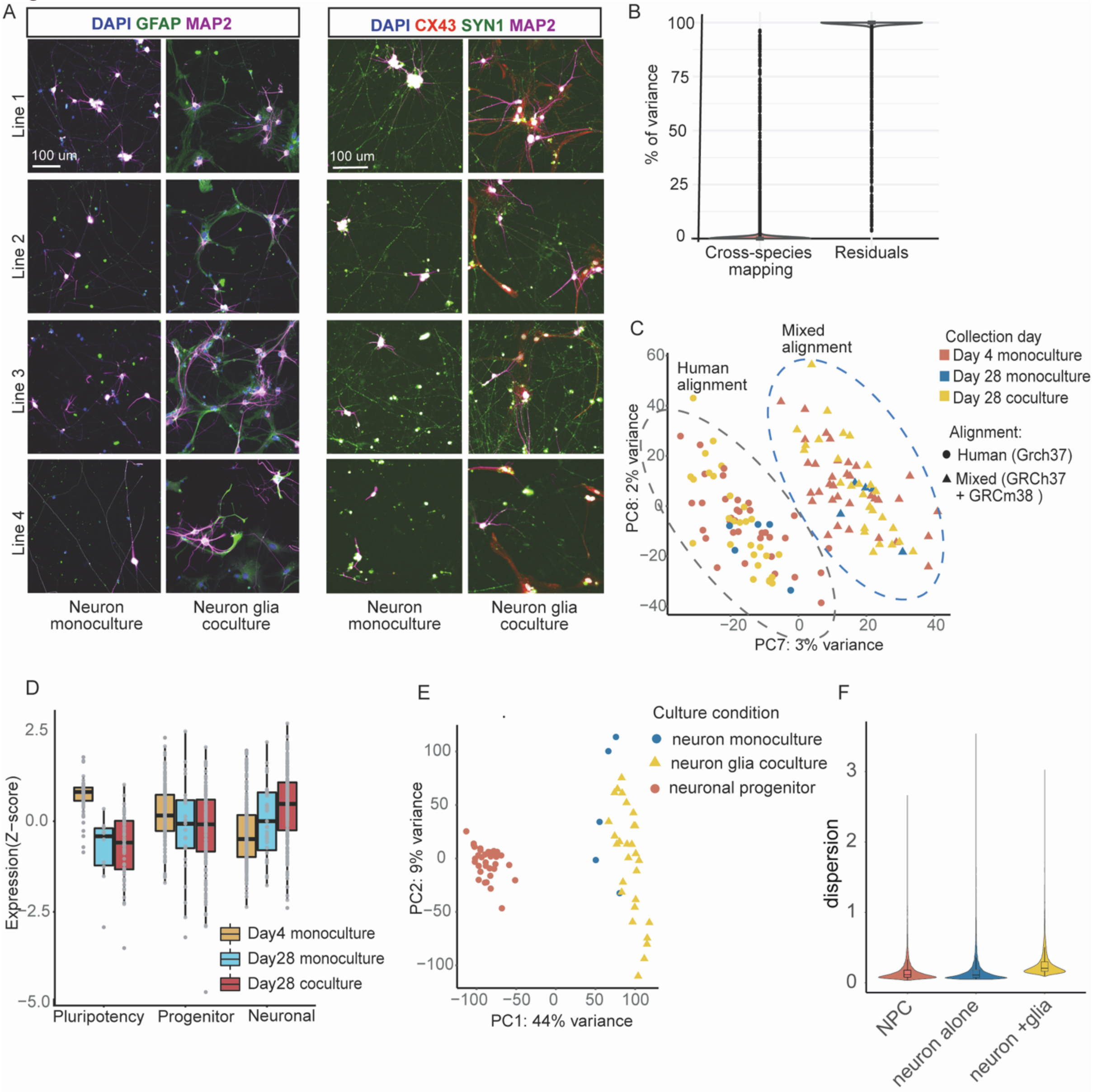

Figure S2. Variance explained by model covariates and GO terms for genes induced by astrocytes in neurons. A) Variance explained in gene expression for model covariates. B) Transcriptome-wide significantly induced genes are enriched for functions in neuronal development and synapse. Related to Figure 3.

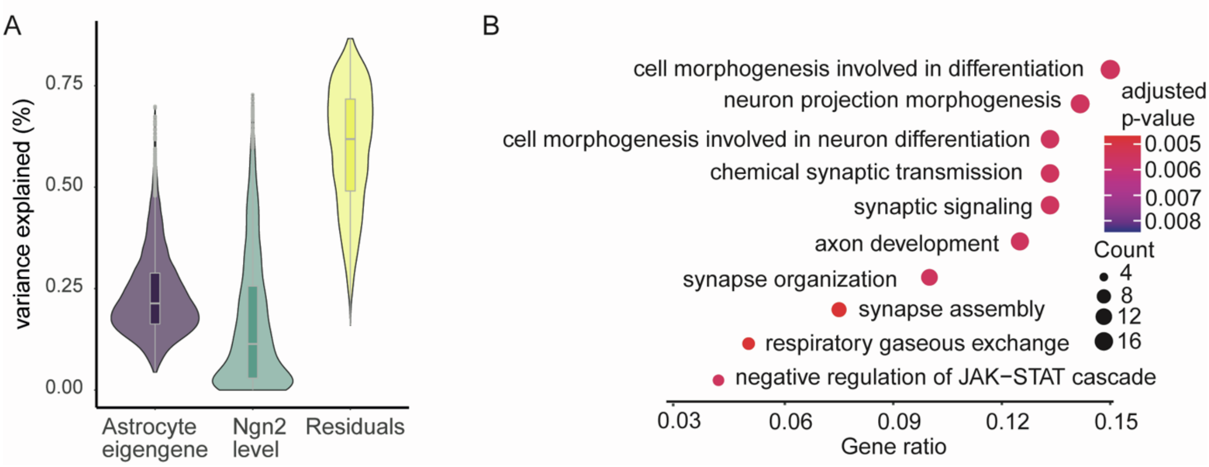

Figure S3. Glial genes that associate with neuronal maturation are predominantly expressed by mouse astrocytes and enriched for synaptic genes. A) tSNE projection of scRNA-seq data showing average expression of 846 genes that were associated with astrocyte eigengene and induced in coculture. B) The 123 positively associated genes had the highest expression in astrocytes in the single cell atlas of adult mouse brain. The 36 negatively associated genes were uniformly expressed across glial cell types in the mouse brain atlas. C) The 159 glial genes have synaptic functions in SynGO. D) Glial genes with negative association to neuron maturation are reduced in astrocytes after contact with neurons. Related to Figure 4 and Figure 5.

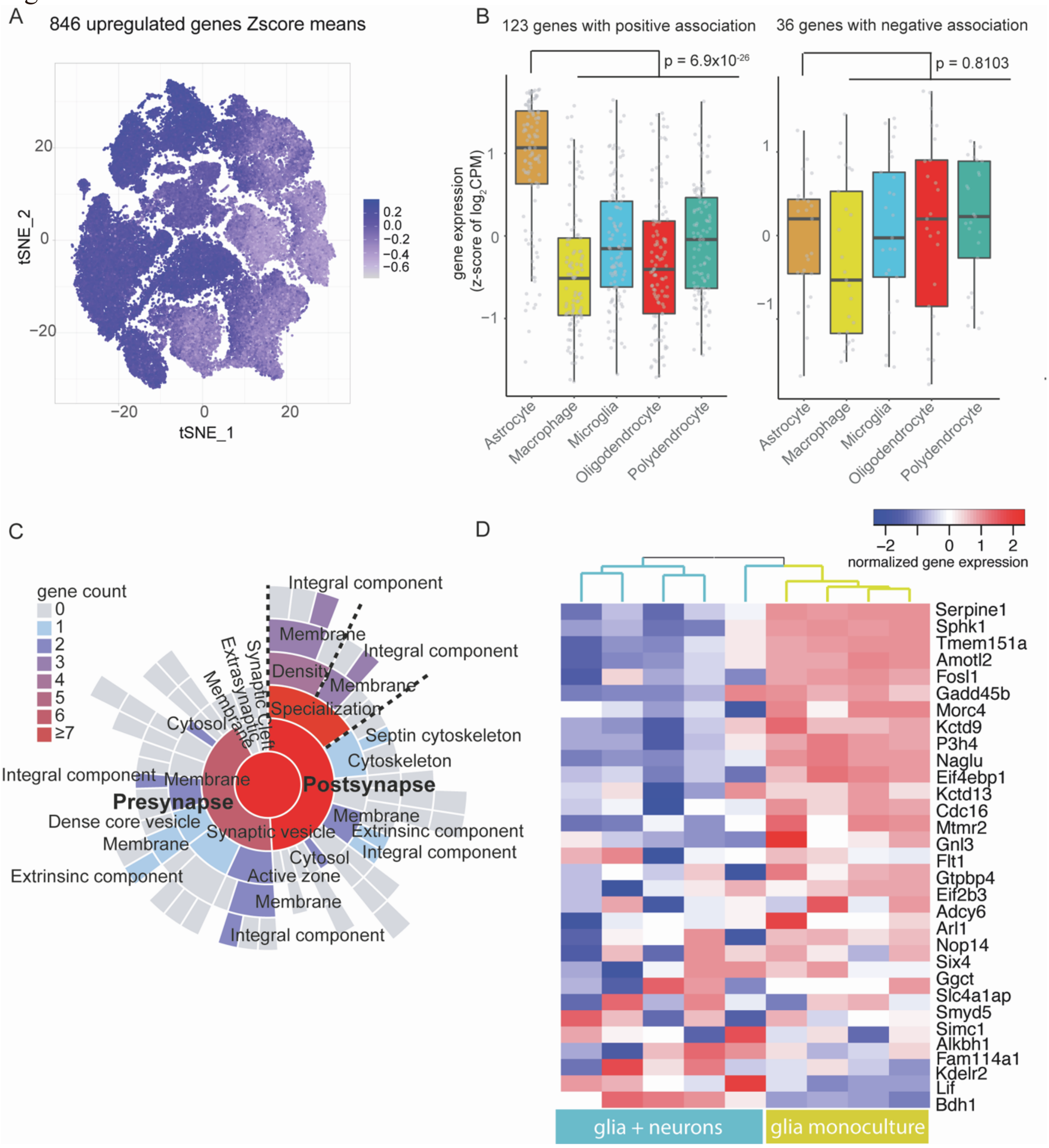

Figure S4. Transcripts encoding members of the cholesterol synthesis pathway are induced in astrocytes after contact with neurons. Related to Figure 5.

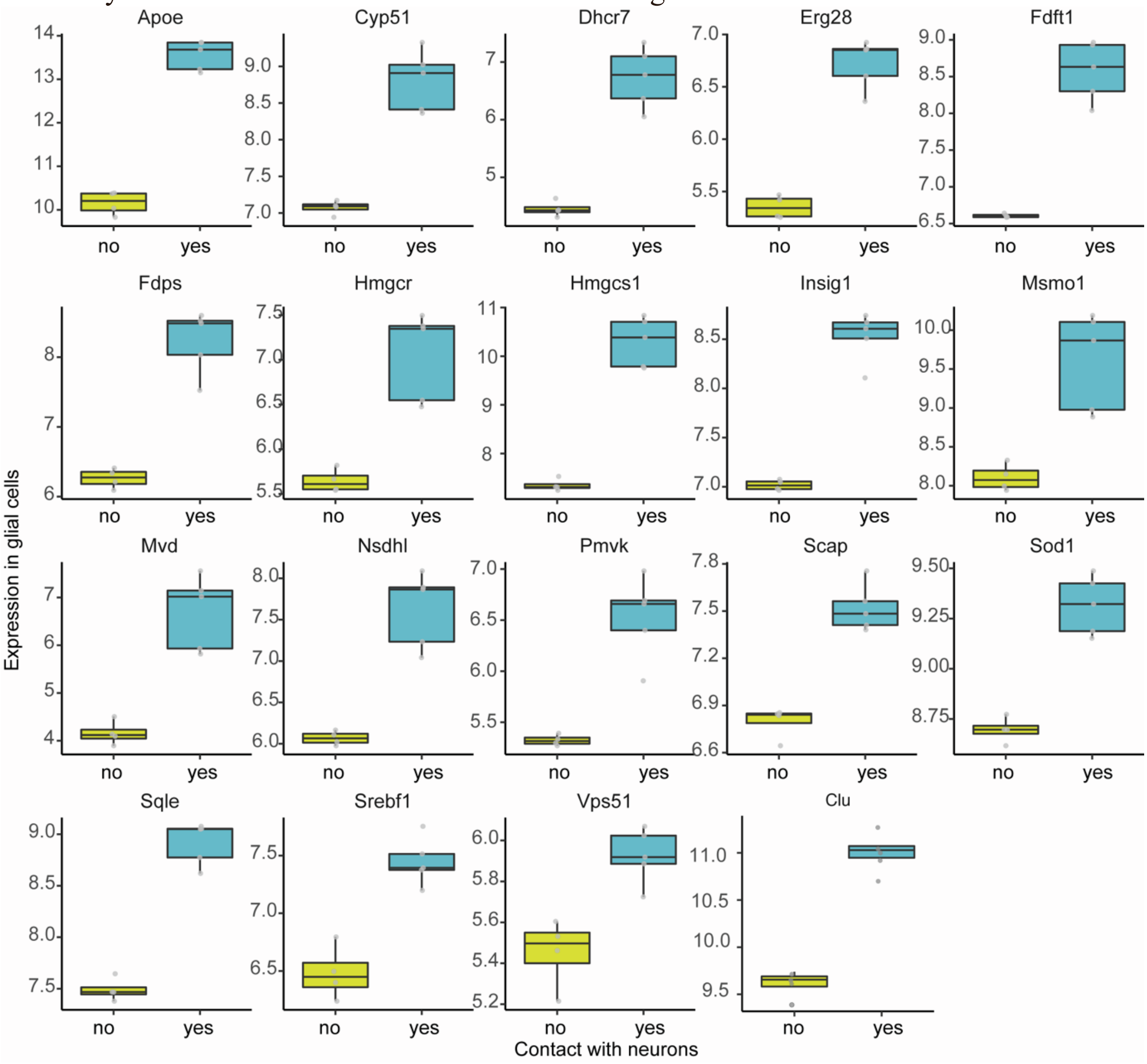

Figure S5. Neurons in sandwich culture and coculture with glial cells have global transcriptional changes. A) Hierarchical clustering of neurons in sandwich and coculture. B) Volcano plot of differential gene expression between neurons in coculture and sandwich culture. C) Neurons in coculture have on average higher expression of genes that associate with the astrocyte eigengene ($p=1.9x10^{-26}$ paired t-test). D) The average levels of the transcripts that associate with the astrocyte eigengene are consistently higher in all neuronal lines. E) Gene-wise associations for 123 glial genes whose high expression induce synaptic gene programs in neurons. The heat-map coloring indicates the standardized z-score of gene-wise p-values from MAGMA. The genes are ordered by the strength of association (z-score value) to schizophrenia. Synaptic genes are colored in red. Genes with direct evidence through coding variants and fine mapping in schizophrenia are indicated by arrows. Genes located in genome-wide significant loci in PGC3 for schizophrenia are highlighted by blue boxes. Related to Figure 7.

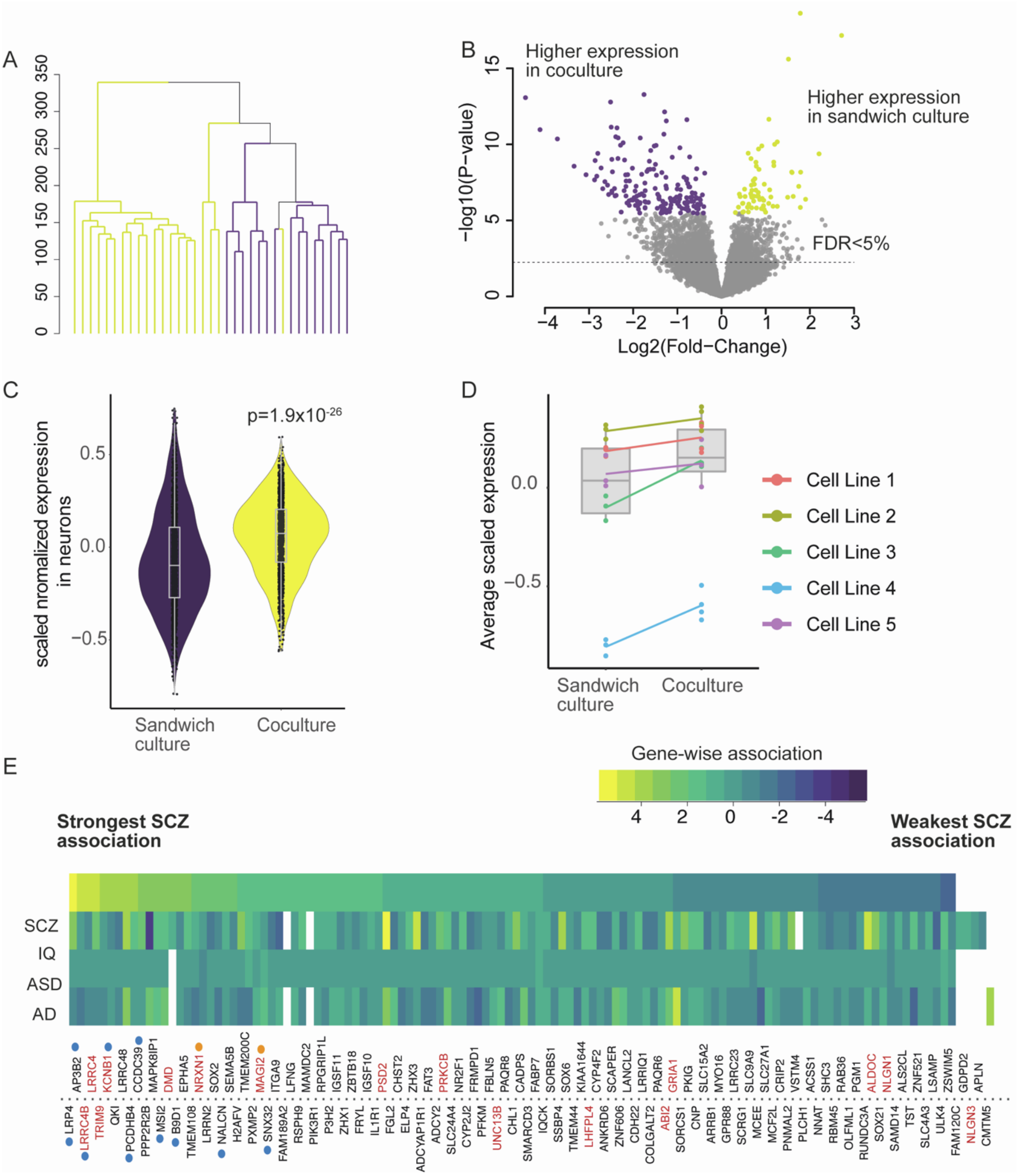
A
0
50
100
150
200
250
300
350
B
Higher expression in coculture
Higher expression in sandwich culture
FDR<5%
−log10(P−value)
Log2(Fold−Change)
C
p=1.9x10-26
scaled nromalized expression in neurons
Sandwich culture
Coculture
D
Average scaled expression
Cell Line 1
Cell Line 2
Cell Line 3
Cell Line 4
Cell Line 5
Sandwich culture
Coculture
E
Gene-wise association
Strongest SCZ association
Weakest SCZ association
SCZ
IQ
ASD
AD
LRP4
AP3B2
LRRC4B
LRRC4
TRIM9
KCNB1
QKI
LRRC48
PCDHB4
CCDC39
PPP2R2B
MAPK8IP1
MSI2
DMD
B9D1
EPHA5
TMEM108
NRXN1
LRRN2
SOX2
NALCN
SEMA5B
H2AFV
TMEM200C
PXMP2
MAGI2
SNX32
ITGA9
FAM189A2
LFNG
RSPH9
MAMDC2
PIK3R1
RPGRIP1L
P3H2
IGSF11
ZHX1
ZBTB18
FRYL
IGSF10
IL1R1
PSD2
FGL2
CHST2
ELP4
ZHX3
ADCYAP1R1
FAT3
ADCY2
PRKCB
SLC24A4
NR2F1
CYP2J2
FRMPD1
PFKM
FBLN5
UNC13B
PAQR8
CHL1
CADPS
SMARCD3
FABP7
IQCK
SORBS1
SSBP4
SOX6
TMEM44
KIAA1644
LHFPL4
CYP4F2
ANKRD6
SCAPER
ZNF606
LANCL2
CDH22
LRRIQ1
COLGALT2
PAQR6
ABI2
GRIA1
SORCS1
PKIG
CNP
SLC15A2
ARRB1
MYO16
GPR88
LRRC23
SCRG1
SLC9A9
MCEE
SLC27A1
MCF2L
CRIP2
PNMAL2
VSTM4
PLCH1
ACSS1
NNAT
SHC3
RBM45
RAB36
OLFML1
PGM1
RUNDC3A
ALDOC
SOX21
NLGN1
SAMD14
ALS2CL
TST
ZNF521
SLC4A3
LSAMP
ULK4
ZSWIM5
FAM120C
GDPD2
NLGN3
APLN
CMTM5
